# Individual Risk Assessment for Population Living on the Territories Long-Term Polluted by Organochlorine Pesticides

**DOI:** 10.3390/toxics11060482

**Published:** 2023-05-25

**Authors:** Aleksandr Garshin, Nazym Altynova, Erika Djangalina, Ozada Khamdiyeva, Gulminyam Baratzhanova, Anar Tolebaeva, Zhasulan Zhaniyazov, Elmira Khussainova, Céline Cakir-Kiefer, Stefan Jurjanz, Matthieu Delannoy, Leyla Djansugurova

**Affiliations:** 1 Institute of Genetics and Physiology, Al-Farabi Avenue, 93, Almaty 050060, Kazakhstanleylad@mail.ru (L.D.); 2 Faculty of Biology and Biotechnology, Al-Farabi Kazakh National University, Al-Farabi Avenue, 71, Almaty 050040, Kazakhstan; 3 INRAE, URAFPA, Université de Lorraine, F-54000 Nancy, Francestefan.jurjanz@univ-lorraine.fr (S.J.); matthieu.delannoy@univ-lorraine.fr (M.D.)

**Keywords:** pesticides, chromosome aberrations, exposure survey, food habits, cytogenetic analysis, molecular genetic analysis

## Abstract

The long-term storage of unutilized pesticides raised new problems of long-term environmental contamination. The study presents the results of surveying 151 individuals in 7 villages living close to pesticide-contaminated localities. All individuals have been surveyed concerning their consumption habits and lifestyle characteristics. An assessment of the general exposure risks of the local population was carried out using the analysis of pollutants in food products and the average levels of their consumption in the region. The cohort risk evaluation revealed that the greatest risk was associated with the regular consumption of cucumbers, pears, bell peppers, meat, and milk. The new model to estimate individual risks of long-term pesticide pollution was proposed as a calculation of the combined action of 9 risk factors, including individual genotypes, age, lifestyle, and personal pesticide consumption rates. The analysis of the predictive ability of this model showed that the final score for individual health risks corresponded to the development of chronic diseases. A high level of chromosomal aberrations was evidenced for individual genetic risk manifestations. The combined influence of all risk factors revealed contributions of 24.7% for health status and 14.2% for genetic status, while other impacts go to all unaccounted factors.

## 1. Introduction

Organochlorinated pesticides (OCP) are chemicals intended to poison the target organisms of pests, mainly insects. As a result, the destruction of biocenoses in areas where pesticides are used is becoming a global problem [1,2]. Under the action of pesticides, some of the biological reactions are impaired, which allows a person to control the spread of plant diseases, preserve food for longer, and destroy pests [1,3]. However, the food products, grown up with pesticide treatment can significantly influence consumer’s health. Therefore, usefulness and safety of such products should be taken into account. 

OCPs are considered toxic to human health. Export, import, and use of the most dangerous OCPs are prohibited in accordance with the decisions of the Stockholm Convention (2001), the fourth meeting of Parties of the Conference (2009), and further redactions of the list of prohibited POPs [4,5] accepted by the majority of countries, including the Republic of Kazakhstan. However, in Kazakhstan, many stocks of pesticides with POP properties were not withdrawn but were widely used in agriculture during 2003–2012 because the territories of pesticide storage facility locations had been privately owned (unofficially recognized by local authorities, personal communication).

According to the United Nations Environmental Program, the statistics concerning obsolete pesticides in Kazakhstan revealed that more than 1500 tons of banned obsolete pesticides and their mixtures of unknown composition were used for agricultural purposes before 2001. According to inventory data for 2009–2010, 64 pesticide storage facilities were detected in the Almaty region of Kazakhstan, where 68,443 kg of obsolete and unusable pesticides was accumulated. Among them, there was 35,953 kg of labeled prohibited pesticides (organochlorine, organophosphorus, simm-triazine, fluorine-containing, and thio-carbamate classes) and 32,550 kg of pesticide mixtures of unknown composition [6,7,8,9,10].

Pesticide exposure (skin and/or inhalation absorption) is known to be associated with numerous harmful effects, with the most frequent being reproductive disorders and carcinogenesis, less frequent manifestations of neurodegenerative and cardiovascular diseases, and even genetic damage and the induction of chromosomal changes [11,12]. Concerning the carcinogenicity of OCPs, epidemiological studies revealed a high prevalence of hematopoietic bone marrow cancer, including myelodysplastic syndrome (MDS), acute myeloid leukemia (AML), and multiple myeloma [12,13]. Associated with pesticide exposure, chromosomal damages have been identified in several populations. Some researchers noticed significant differences in the frequency of chromosomal aberrations in exposed individuals compared to unexposed controls [14,15,16,17], but others did not confirm such an association [18]. These contradictions may be due to the fact that unstable chromosomal aberrations (dicentrics, acentrics, interstitial deletions, etc.) are eliminated in the process of cell proliferation. The mutagenic effect of pesticides, as measured by the unstable chromosomal aberration rate in blood lymphocytes, can be detected only 2–3 months after pesticide exposure, before cell division. We suppose that in some cases of cytogenetic analysis of populations chronically exposed to pesticides, researchers could not take into account the time difference between the date of individual pesticide consumption and the time of blood sampling. Therefore, the level of unstable chromosome aberration cannot be used as a reliable health risk index.

The mutagenic effect of pesticides can also be influenced by the expression of a number of genes that regulate the metabolism of toxicants and DNA repair. Significant associations of mutations and polymorphisms of genes involved in DNA repair (*XRCC1*, *XRCC3*, *XPD*, etc.), detoxification of xenobiotics (*GSTM1*, *GSTT1*, *GSTP1*, *CYP1A1*, etc.), and antioxidant protection (*SOD1*, *GCLC*, *GCLM*, *GPX4*, *NFE2L3*, etc.) with the development of a number of multifactorial diseases [19,20] are well known. The genetic polymorphisms of these genes can modulate susceptibility to pesticide exposure and serve as determinants of toxicity [21]. The genetic status of these genes should be considered when assessing the toxic effects of pesticides in populations exposed for long periods of time to environmental pesticide contamination.

Human health risk assessment (HHRA) is the process of assessing the nature and likelihood of adverse human health effects on the health of humans who may be exposed to chemicals in contaminated environmental media, now or in the future.

The earliest risk assessment methods were based on studies on laboratory animals (mice, guinea pigs, chickens, etc.), with subsequent extrapolation to humans [22]. However, such extrapolation may be unreliable in humans due to differences in ecological environments and the variability of individual factors, such as age, heredity, and the presence of other risk factors such as smoking, etc.

Currently, the most common approach in scientific research to assess the risks of exposure to pesticide contamination is the method of calculating the hazard quotient (HQ) and hazard index (HI), which was first proposed by the US Environmental Protection Agency (EPA). Some studies of risk evaluation for pesticide toxicity offer alternative approaches [23] or modifications of the HHRA method [24], considering the limitations of this risk assessment method.

The calculation of HI and HQ makes it possible to theoretically assess the short-term and long-term risks to the health of the population exposed to pesticides, as well as to determine the most dangerous groups of pesticides in a contamination cocktail. These values are calculated from the difference between the acceptable daily intake (ADI), defined as the maximum amount of a chemical that can be consumed daily over a lifetime without appreciable health risk [25], on the one hand, and the estimated daily intake (*EDI*) on the other hand.

Although this method is accepted for evaluating health risks, it does not consider the individual variables of each person, only evaluating the average values of body weight and food consumption in the population. Since consumption rates and individual characteristics are different for each person, to assess the risk of exposure to pesticides, we aim to assess the contribution of innate and environmental factors, as well as dietary habits.

It is well known that bad habits such as smoking and consequent alcohol consumption also affect human health [26,27]. Although their influence on the severity of the toxic effect of pesticides is still not thoroughly investigated [28,29], adding this factor to the risk assessment might also provide more detailed results.

The most advanced risk assessment protocols for various classes of chemicals include identifying the most sensitive groups of people by measuring a wide range of individual characteristics such as age, sex, ethnicity, location, social and economic status, lifestyle, and personal diet (EFSA and EPA protocols). However, this approach is still not common in the assessment of organochlorine pesticide risk to human health.

Therefore, this study aims to suggest a combined approach to assess the health risk for the population chronically exposed to pesticides in the food chain, accounting for all available data and possible risk factors. The purpose of this study was to evaluate the risk of long-term pesticide influence on cohort and individual levels using the example of residents in different villages having an exposure context in the Almaty region of Kazakhstan. For the estimation of the input of possible risk factors to health and genetic risk, we propose a method for calculating risks, taking into account the following data: the amount of pesticides and heavy metals consumed in food, health, and cytogenetic data; the state of the main genetic systems involved in protecting the body from the effects of toxicants and DNA repair; and individual physiological and lifestyle characteristics (age, sex, weight, smoking, and alcohol consumption).

## 2. Materials and Methods

### 2.1. Objects and Materials

The collection of biomaterials (peripheral blood samples, samples of food products of plant and animal origin) was carried out in 5 settlements of the Talgar region (Beskaynar, Kyzylkairat, Amangeldy, Belbulak, and Enbekshi settlements) in the period of 2018–2021 years and in 2 settlements of the Dzhambyl region (Karakastek and Umbetaly settlements) in 2021. The burials of obsolete, non-utilized pesticides were found on the territory of each selected village.

**Figure 1:**
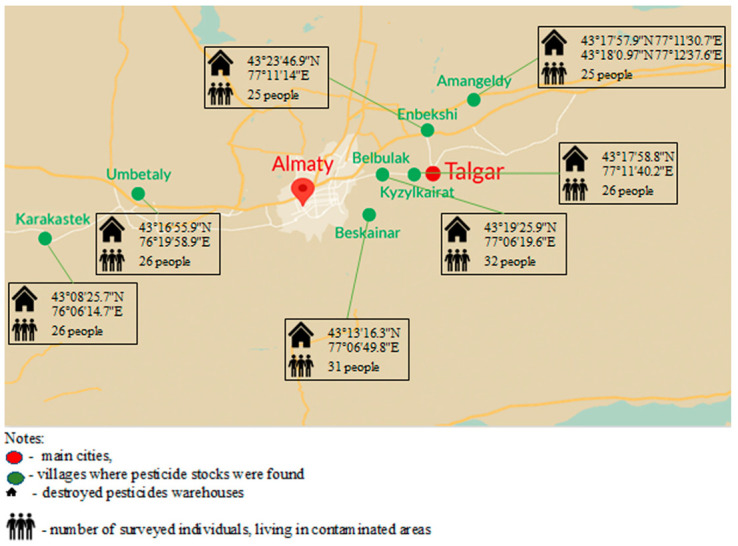
Location of destroyed pesticide warehouses and surveyed cohorts.

The material for cytogenetic and molecular genetic analysis was samples of peripheral blood taken from people living in close proximity to warehouses of unutilized pesticides, only after obtaining voluntary informed consent to the study (Figure 1).

Protocol of the Local Ethics Commission of the PEO Kazakh-Russian Medical University (No. 52, dated 9 May 2017) and Protocol of the Local Ethics Commission of the RSE “Institute of Human and Animal Physiology”, SC MES RK No. 6, dated 7 December 2020, provided the initial and further questioning, collections of peripheral blood samples, and published study results.

### 2.2. Questioning

The data on age, ethnicity, sex, habits, and health status were collected between 2018 and 2022 when peripheral blood samples were taken from 191 people. In the period from 2020 to 2021, additional questioning of 151 residents of the surveyed villages was conducted by the face-to-face interview method. Questionnaires included detailed data about personal food consumption habits (kind and amounts of consumed foodstuffs per day, how regularly they were consumed, sources of food products of plant and animal origin, questions about health status, length of residence, etc.). All the collected data were used later for the estimation of individual daily intakes of each food type, as well as the assignment of health status and age groups for people. An example of a detailed questionnaire is provided in the Supplementary Materials (Supplementary Materials, Figure S1).

### 2.3. DNA Isolation

Genomic DNA was isolated from frozen (−20 °C) peripheral blood samples using commercial kits, “Genomic DNA Purification Kit” and “Gene Jet DNA Purification Kit” (ThermoFisher, Waltham, MA, USA). Quantitative and qualitative evaluation of the isolated DNA samples was performed using spectrophotometric (BioPhotometer Plus, Eppendorf, Germany; NanoDrop 2000, Thermo Fisher Scientific, Waltham, MA, USA) and photometric (Qubit Fluorometric Quantification, Thermo Fisher Scientific, Waltham, MA, USA) systems.

### 2.4. Genotyping by Multiplex PCR and PCR-RFLP

The PCR-RFLP method was used for genotyping polymorphic regions of detoxification genes (*GSTT1* (rs1601993659), *GSTM1* (rs1183423000), and *GSTP1* Ile105Val (rs1695)), DNA repair genes (*XRCC1* Arg194Trp (rs1799782), *XRCC1* Arg399Gln (rs25487), *XRCC3* Thr241Met (rs861539), and *XPD* Lys751Gln (rs13181)), and antioxidant protection genes (*GCLC -129C/T* (rs524553), *GCLM -588C/T* (rs41303970), and *GPX4 718CT* (Leu220Leu) (rs713041). To set up PCR-RFLP, a preliminary design of primers specific for the studied regions of the *SOD3* and *GPX4* genes was performed. The design was performed using the online application “Basic Local Alignment Search Tool (primer-BLAST)” developed by the National Center for Biotechnology Information (NCBI). When constructing, genome-wide RefSeq sequences were taken as a basis, including the *SOD3* and *GPX4* genes (Homo sapience), obtained from the nucleotide database of the National Center for Biotechnology Information (NCBI RefSeq nucleotide database).

For the *GCLC* and *GCLM* genes, ready-made primer sequences developed by Shun-ichi Koide et al. [30] for the *GCLC* (F: 5′TCGTCCCAAGTCTCACAGTC 3′; R: 5′CGCCCTCCCCGCTGCTCCTC 3′) and Shin-ichi Nakamura et al. [31] for the *GCLM* (F: 5′CTCAAGGGCAAAGACTCA 3′; R: 5′CCGCCTGGTGAGGTAGACAC 3′) were used.

Primers were synthesized on an ASM-800 synthesizer (OOO Biosset, Russia). After cleaning and lyophilization, the resulting primers were diluted in 200 µL ddH_2_O and frozen at −20 °C for further use.

Extended deletions in the *GSTT1* and *GSTM1* genes were analyzed by multiplex PCR. For the *GSTM1* and *GSTT1* genes, two genotypes were determined: homozygous for the deletion («–/–») and positive, i.e., carrying a functional allele in a homozygous («+/+») or heterozygous state («+/–»). Analysis of the polymorphic loci of the *GSTP1*, *XRCC1*, *XRCC3*, *XPD*, *GCLC*, *GCLM*, and *GPX4* genes was performed by polymerase chain reaction with restriction fragment length polymorphism analysis (PCR-RFLP). Visualization of amplification and restriction products was carried out by gel electrophoresis in 3% agarose gel.

### 2.5. Genome-Wide Microarray SNP Genotyping and Bioinformatics Processing

The preparation of DNA samples for microchip genotyping was performed using the Infinium Automation Kit-8 Tip Tecan Non-LIMS automated sample preparation station. Genome-wide microchip genotyping of target population cohorts was carried out on the iScan System (Illumina, San Diego, CA, USA) platform using biochip kits (Infinium^®^ ImmunoArray-24 v2.0 BeadChip Kit and Infinium Global Screening Array-24 Kit, Illumina, San Diego, CA, USA) according to the Infinium HTS Automated Workflow protocol.

The original microarray genotyping data were processed using Illumina GenomeStudio v.2.05 software (Illumina, San Diego, CA, USA), PLINK (Purcell, S.; Neale, B.; Todd-Brown, K.; Thomas, L.; Ferreira, M.A.R.; Bender, D.; Maller, J.; Sklar, P.; de Bakker, P.I.W.; Daly, M.J. & Sham, P.C. PLINK: a toolset for whole-genome association and population-based linkage analysis. *American Journal of Human Genetics*, **2007**, 81.), RStudio (RStudio Team (2020). RStudio: Integrated Development for R. RStudio, PBC, Boston, MA URL http://www.rstudio.com/ (accessed on 10 June 2022). Samples with a quality of less than 98% (the percentage of genotyped SNPs, expressed in call rates) were excluded from the analysis.

Bioinformatic analysis of the results of microchip SNP genotyping included the determination of the reliability of the data, the determination of the homozygosity/heterozygosity for the identified SNPs, and the detection and analysis of the pathogenicity of the identified mutations and polymorphisms by comparing the found mutations with mutations identified as pathogenic in known databases and scientific articles. Genome-Wide Association Studies data catalog (GWAS catalog—https://www.ebi.ac.uk/gwas/) (accessed on 5 June 2022), database of single nucleotide polymorphisms (dbSNP, https://www.ncbi.nlm.nih.gov/snp/) (accessed on 5 June 2022), ClinVar (https://www.ncbi.nlm.nih.gov/clinvar) (accessed on 5 June 2022), and 1000 Genomes (1000 G—https://www.internationalgenome.org/1000-genomes-browsers) (accessed on 5 June 2022).

### 2.6. Statistical Methods

The results obtained were processed by traditional statistical methods, including the estimation of average values and standard deviations, T statistics, and the estimation of *p*-values. Cytogenetics data were taken from our earlier publication on the topic [32]. Differences were declared significant at *p* < 0.05. The significance level (*p*) was determined using Chi2 (χ^2^) and Student’s *t*-test.

The genotype distribution for single nucleotide variants was estimated by matching to Hardy–Weinberg equilibrium (HWE) with 2 degrees of freedom. Variants deviating from HWE with *p* < 0.05 were considered statistically significant.

#### 2.6.1. Calculation of Short-Term and Long-Term Risks for Villages

After analyzing all the latest data on methods for determining the risk of pesticide pollution [33,34,35,36,37,38,39,40], we took the following approaches, operating with an average indicator as the basis for calculating individual risk. The estimated daily intake (*EDI*) of pesticide residues found in different food samples is calculated for each age category using the equation below.

$$EDI=\frac{C*IR*EF*ED}{BwxAT},$$

where *C* is the mean concentration of contaminants (mg/kg), *IR* is the rate of ingestion of food (kg/day), *EF* is the frequency of exposure (365 days/year), *ED* is the duration of exposure (days), *Bw* is the average body weight (kg) calculated from questionnaire data, and AT is the time over which the dose is averaged (in days).

An AT of 70 years (25,550 days) was considered to evaluate the carcinogenic risk.

To simplify the risk assessment, all pesticides that were detected in food products were divided into six groups depending on the chemical structure of the active POPs: (1) DDT group (4.4 DDT; 4.4-DDD; 2.4-DDD; 4.4-DDD); (2) HCB group (hexachlorobenzene, hexabromobenzene); (3) hexachlorocyclohexane group (α-HCH, γ-HCH, β-HCH, δ-HCH); (4) aldrin group (aldrin, endrin, deleldrin, endrin aldehyde); (5) endosulfan group (endosulfan 1, endosulfan 2, endosulfan sulfate); and (6) heptachlor group (heptachlor, heptachlor epoxide).

The calculation of MPC excess was performed on the basis of EU and Customs Union MPCs (Supplementary Materials, Table S2). MPC excess was calculated for each group of pesticides and for each food item.

The hazard quotient, which is an assessment of the short-term risk, is calculated by the formula:

$$HQ=\frac{EDI}{ADI}$$

The hazard index reflects the assessment of long-term risk and is calculated by the formula:

$$HI=\frac{EDI}{ADI}*100$$

The estimated daily consumption of pesticides is most often calculated from the residues of toxic substances found in products and the daily consumption of these products. Since body weight also affects tolerance to various types of toxic substances, this factor is also taken into account in the final equation:

$$EDI=\frac{C*Cons}{Bw},$$

where *C* is the quantity of a toxic substance in 1 kg (mg/kg). of the product, *Cons* is the average daily consumption of the product in the given area (kg/day), and *Bw* is the body weight (kg) [34].

The acute/short-term *HQ* assessment (*aHQ*) was calculated based on the estimated short-term intake (*ESTI*) and the acute reference dose (*ARfD*), using the following equation:

$$aHQ=\frac{ESTI}{ARfD}*100\%$$

Estimated short-term intake (*ESTI*) is calculated using the equation below:

$$ESTI=\frac{\Sigma F\;x\;\mathrm{HR}:P}{mean\;body\;weight}\;,$$

where *F*—full portion consumption data for the commodity unit and HR:P—the highest residue level.

Chronic/long-term HQ assessment (*cHQ*) is calculated based on the estimated daily intake (*EDI*) and the acceptable daily intake (*ADI*) [18], using the following equation:

$$cHQ=\frac{EDI}{ADI}*100\%$$

Estimated daily intake (*EDI*) is calculated using the equation below:

$$EDI=\frac{C*CR}{Bw},$$

where *C* is the mean concentration of pesticide residues (mg/kg), *CR* is the consumption rate (kg/d), and *Bw* is the average body weight (kg).

Information on ARfD and *ADI* is obtained from the JMPR, available at: http://apps.who.int/pesticide-residues-jmpr-database/Home/Range/All (accessed on 7 October 2021).

Hazard identification determines whether exposure to a pesticide is likely to increase the incidence of specific adverse health effects. The hazard description aims to quantify the relationship between dose level and morbidity. Risk assessment compares the potential intake or consumption of pesticide residues with *ADI* or *ARfD* [34,35,36].

To characterize the non-carcinogenic risk, the hazard index (*HI*) coefficient is used, which is calculated as the ratio of the *EDI* to the acceptable daily intake (*ADI*) according to the formula below:

$$HI=\frac{EADI}{ADI}$$

If the coefficient is less than 1.0, then there is practically no probability of an adverse impact. However, if the ratio exceeds 1.0, then the food is considered hazardous to the health of consumers [39].

#### 2.6.2. Calculation of Individual Risks

To increase the efficiency of risk calculation, we proposed improving the risk calculation formulas by introducing additional criteria and evaluating individual risks using the multiple regression method. The method of multiple regression is widely used in medicine to determine the degree of influence of many independent factors on the risk of developing a particular disease. Despite the fact that this method shows high efficiency in risk assessment, its use in studies on the effects of toxic substances in the environment is still not widespread.

The multiple regression equation can be represented as follows:

$$Y=f\left(\beta \;,\;X\right)+\varepsilon ,$$

where *X* = *X* (*X*_1_, *X*_2_,…, *X_m_*) is a vector of independent (explanatory) variables; *β*—vector of parameters (to be determined); *ε*—random error (deviation); and *Y*—dependent (explained) variable.

The theoretical linear multiple regression equation is:

$$Y=\beta_{0}+\beta_{1}X_{1}+\beta_{2}X_{2}+\ldots +\beta mXm+\varepsilon ,$$

where *β*_0_ is a free term that determines the value of *Y*, in the case when all explanatory variables *Xj* are equal to 0.

Data on product contamination level, cytogenetic analysis, and health status of individuals exposed to pesticide contamination were published earlier [32]. We used published data to calculate individual risks.

Genetic status (frequency of aberrations) and health status (diagnosed diseases) were chosen as dependent variables (Y). The risk calculation for both variables was carried out separately. As independent variables (X), the risk factors presented in the equation were selected: age (X_1_), exceeding the permissible levels of pesticides (X_2_ and X_3_), and the permissible level of heavy metals (X_4_) in food products, as well as smoking (X_8_) and alcohol consumption (X_9_). The equation also included indicators of the functional state of the genetic system of DNA repair with candidate genes *XRCC3*, *XRCC1*, *XPD* (X_5_), detoxification of xenobiotics with candidate genes *CYP1A1*, *CYP2B6*, *CYP2D6*, *CYP2C19*, *GSTP1*, *GSTT1*, *GSTM1* (X_6_), and antioxidant protection with candidate genes *NFE2L3*, *SOD1*, *GCLC*, *GCLM*, and *GPX4* (X_7_). To be included in the multiple regression equation, a scoring system for assessing the state of genes for each of the genetic systems was introduced. The score for each gene ranged from 0 to 1, where 0 is the full functionality of all relevant proteins and the absence of risk, and accordingly, the absence of risk, and 1 is the presence of a non-functional allele in the gene and the presence of risk.

Using the collected data, the amount of daily consumption of food products was evaluated for each type of food (meat, cucumbers, tomatoes, peppers, apples, pears, and milk). Based on the chemical analysis of the pesticide contents of each type of food, the maximum theoretical daily intake of pesticides for each individual was estimated using the formula:

$$EDI=C*\frac{Cons}{Bw},$$

where *C* is the estimated concentration of a toxic substance in 1 kg of the product, *Cons* is the estimated daily consumption of the product for each individual, and *Bw* is the body weight of each individual, taken from questionnaire data. Smoking and drinking factors were also included in the questionnaire.

The estimation of pesticide and heavy metal levels was performed for seven types of food products (meat, cucumbers, tomatoes, peppers, apples, pears, and milk), six groups of pesticides (DDT, HCB, HCH, aldrin, endosulfans, and heptachlor), and eight types of heavy metals (Cu, Zn, Ni, Co, As, Pb, Cd, and Cr).

The health status and percentage of chromosomal aberrations were taken as two separate dependent variables *Y*, with each calculated separately in the multiple regression equation.

A ranking system depending on the number of registered chronic diseases was introduced for health status assessment, where 0 was the absence of diseases, 1—one chronic disease, 2—two chronic diseases, etc.

To calculate the degree of influence of each factor on the dependent variable *Y*, a vector of estimates of regression coefficients was used.

To calculate the statistical significance of each regression coefficient, the following equation was applied:

$$t_{i}=\frac{b_{i}}{S_{bi}}$$

If the final value exceeded the calculated number of degrees of freedom (2.263), the regression coefficient was considered significant.

The closeness of the joint influence of all factors on the dependent variables was assessed using the coefficient of determination:

$$R^{2}=1-\left(1-R^{2}\right)*\frac{n-1}{n-m-1}$$

The closer this coefficient is to one, the more the regression equation explains the behavior of *Y*. The value of this coefficient, multiplied by 100, determines the percentage of variability of the studied risks under the influence of all factors.

To assess the predictive ability of the resulting formulas for calculating risks, the final value of the formulas was used to calculate the arithmetic mean, which was compared with the known value of the independent variables *Y* (health status and the number of aberrations). Values above the arithmetic mean were considered an indicator of higher risk, and vice versa, values below the arithmetic mean indicated no risk.

## 3. Results

### 3.1. Questioning Results

A total of 151 people were interviewed in detail, all of whom lived in close proximity to pesticide storage. For each of the respondents, individual eating habits (the amount and type of food consumed per day) were obtained, as well as information on chronic diseases, bad habits, and sources of food. According to the survey, the dietary habits of the population differ by village, which can make it difficult to calculate real risks when using known average values. Table 1 presents data on the ethnicity, sex, and age of the surveyed population cohorts.

The medical status of individuals has been evaluated using both clinical examinations and questioning. Cardiovascular diseases (hypertension, ischemia, atherosclerosis, etc.) were prevalent (38 persons, 25.17%). The second most common type was chronic respiratory diseases, identified in 10 respondents (6.62%). Five persons had a history of cancer (3.31%), single cases of kidney disease, thyroid disease, and diabetes were recorded, and nineteen individuals (12.58%) were characterized by different complaints (headaches, joint pain, abdominal pain, surges in blood pressure, fatigue, cognitive and sleep disturbances, weak immunity, etc.).

Because this population lives and consumes food products from areas contaminated by pesticides, we treated the personnel questionnaires regarding information about individual diets and food habits. Table 2 shows the average values of the consumption of the types of products analyzed for the content of pesticides and heavy metals for each of the studied settlements.

Body weight values are necessary for the evaluation of the *EDI* index, which is very important for general and individual risk assessments. It is known that organochlorine pesticides can also accumulate in adipose tissue [41,42,43], which may justify considering body weight as one of the risk factors.

Average data on body weight and consumption of products of plant and animal origin for the region, provided in Table 3, were used to calculate short-term and long-term risks for each cohort.

### 3.2. Genotyping Results

We have analyzed the polymorphisms in genes involved in DNA repair (*XRCC1*, *XRCC3*, *XPD*, etc.) [44,45], detoxification of xenobiotics (*GSTM1*, *GSTT1*, *GSTP1*, *CYP1A1*, *SOD1*, etc.) [46,47], and antioxidant protection (*GCLC*, *GCLM*, *GPX4*, *NFE2L3*, etc.) [48,49,50] that were strongly associated with a number of multifactorial diseases and pesticide toxicity [19,20]. Most polymorphisms are single nucleotide variations (SNV). But we also included well-known deletion polymorphisms in genes encoding the glutathione-S-transferases of the M1 and T1 types. The summarizing results of genotyping populations that are chronically exposed to pesticides are presented in Table 4.

For the deletion polymorphism of the *GSTM1* and *GSTT1* genes, the heterozygous genotypes (+/−) were detected in combinations with normal alleles homozygous (+/+) and “null” genotypes (−/−)—separately. Genotyping results for some SNVs (rs1799782, rs13181, and rs524553) did not show statistical significance. The probable reasons for low levels of certain genotypes are mixed ethnicity and small cohort sizes.

In the general cohort, the obtained allele frequencies for genes involved in organochlorine pesticide biotransformation were at the expected levels that characterize mixed Asian-European populations. Increased frequencies of mutant alleles were observed for the *XRCC1* Arg399Gln (rs25487), *SOD1* (rs1041740, rs138002121), *CYP1A1* (rs17861084), *CYP2B6* (rs8192718), *CYP2D6* (rs186133763), *GSTP1* (rs1138272), and *GCLC* (rs12524550) genes compared with the average values for the population of similarly mixed ethnicities. The frequency of the rare *GSTP1* gene allele (rs1871042) for the studied sample also slightly exceeded the average for the population. The frequency of the *GCLC* polymorphic variant (rs524553) was lower than the population average.

Molecular genetic analysis revealed an increased frequency of non-functional alleles of glutathione-S-transferases M1 and T1, which may have an impact on the decrease in xenobiotic detoxification functions in the examined population and health.

### 3.3. Analysis of Pesticides Intakes

One of the most important factors in the evaluation of pesticide toxicity risk is the calculation of the amount of organochlorine pesticides and their decay products that enter the human body with food. Previously published data on the content of pesticides in food [32] made it possible to calculate this amount both from average indicators based on chemical analysis of food products and from average food consumption (Table 5). Based on the data about individual eating habits that have been obtained during the recent survey, the data about real pesticide intake were also calculated (Table 6).

As seen in the Table 7, the average values of the actual impact of pesticides, with dietary habits taken into account, differ in each studied cohort but are not statistically significant. The real pesticide intake is lower than the estimated average for all pesticide groups. Therefore, it was assumed that an individual approach to assessing the health risks would give a more accurate result.

### 3.4. Assessing of Long-Term and Short-Term Risks

**Figure 2:**
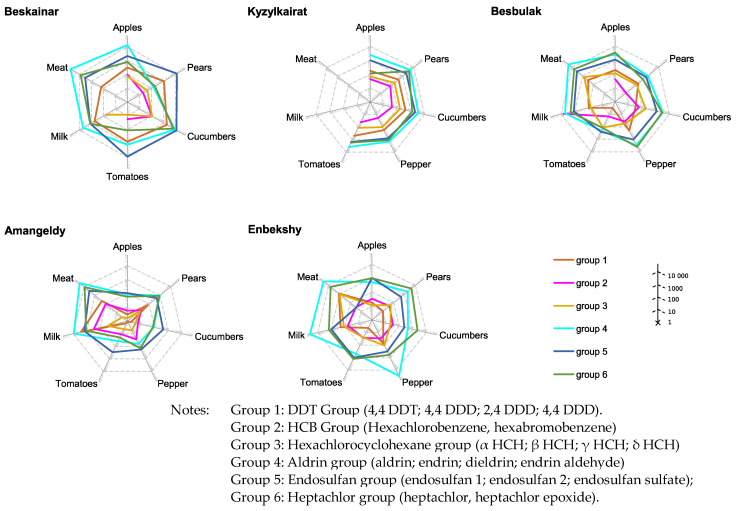
Short-term health risk for population living in close proximity to the sites of unutilized and obsolete pesticide stocks and consuming food contaminated by POPs.

To assess the health risks for surveyed cohorts living in areas contaminated by organochlorine pesticides, we have used the preliminary obtained data on chemical analysis of 24 organochlorine pesticide contents in main food products of plant (apples, pears, bell peppers, tomatoes, and cucumbers) and animal (milk, meat) origin that were grown in contaminated areas [32]. The average for cohort pesticide intakes was calculated using standard food intake norms and detected pesticides in food products, which were divided into six groups depending on the chemical structure of active POPs (Table 5). The acute (short-term) and chronic (long-term) risks of the impact of pesticides on the health of residents of five localities in the Almaty region were determined. (Figure 1 and Figure 2). The calculation of the overall risks for the Karakastek and Umbetaly localities was not effective due to the low content of organochlorine pesticides in food products.

This made it possible to identify types of products and corresponding pesticide groups that may pose the greatest danger to the cohort of the population consuming these products for a long time, contributing to the development of chronic diseases from both a short- and long-term perspective.

The greatest risk is associated with the consumption of pears, cucumbers, bell peppers, milk, and meat since these products have an unacceptably high content of pesticides of the aldrin group (group 4), endosulfan (group 5), and heptachlor (group 6) and are characterized by a high hazard index. In the village of Enbekshi, the hazard ratio exceeds 20%, especially in bell peppers and milk, indicating a high long-term risk of exposure to pesticides.

### 3.5. Assessing of Individual Health Risks

To assess individual health risks, we introduced additional criteria evaluated using the multiple regression method. Instead of average pesticide intake, which was calculated using standard food intake norm values, we have applied individual pesticide intake rates, obtained from real data on personal food consumption and pesticide contents in food. Additional criteria also included age, smoking, alcohol consumption, existing health status, level of chromosomal mutations, and the genetic status of key genes involved in the biotransformation of organochlorine pesticides. Thus, all indexes, including body weight, were personalized to calculate individual health risks. A special ranking system was introduced to define the indexes of health status and genetic status. Individual health status was assessed, taking into account the number of registered chronic diseases.

All studied gene variants (21 polymorphisms of 14 genes participating in pesticide metabolizing and biotransformation that are included in Table 4) were divided into 3 gene clusters in accordance with their functional activities: (a) DNA repair genes—4 SNPs of 3 genes (*XRCC3*, *XRCC1*, *XPD*); (b) genes encoding enzymes of xenobiotic detoxification—9 polymorphisms of 7 genes (*CYP1A1*, *CYB2B6*, *CYP2D6*, *CYPC19*, *GSTT1*, *GSTM1*, *GSTP1*); (c) genes of the antioxidant protection system—8 SNPs of 5 genes (*SOD1*, *NFE2L3*, *GPX4*, *GCLM*, *GCLC*). Each mutant variant predisposing to health risk was assessed at 1 point. The mean index for each cluster, as an indicator of gene system functionality, was included in the formula for individual risk calculation.

Taking into account all included modifications, we propose the following formula for the multivariate analysis of health risks:

Y=0.2975+0.0038∗X1−0.0336∗X2+0.8601∗X3−0.282∗X4+0.09607∗X5+0.5485∗X6+0.2119∗X7−0.3557∗X8+0.1533∗X9

Assessment of separate individual indexes of the formula for individual health risk assessment revealed that the most influential factors on health status are the excess of pesticides in food (influence coefficient = 0.8601) and the state of the xenobiotic detoxification system (influence coefficient = 0.5485). *t*-test confirms the significance of both pesticide and heavy metal excesses when calculating the health risks (T_3_ = 2.562 (>2.263), T_4_ = 2.962 (>2.263)).

We also propose the following formula for the multivariate analysis of genetic risks:

Y=2.4756−0.01415∗X1+0.7166∗X2−0.8569∗X3−0.1146∗X4+1.1351∗X5+0.2174∗X6−0.7496∗X7+0.9268∗X8+0.2767∗X9

The most significant factors affecting the mutation risk were the level of pesticide contamination in food (influence coefficient = 0.8569), smoking (influence coefficient = 0.9268), and the functional state of the DNA repair system (influence coefficient = 1.1351). However, the reliability of the state of DNA repair genes factor is not confirmed (T_5_ = 1.905 (<2.693)), while the factor of pesticide contamination is confirmed (T_2_ = 3.028 (>2.693)).

**Figure 3:**
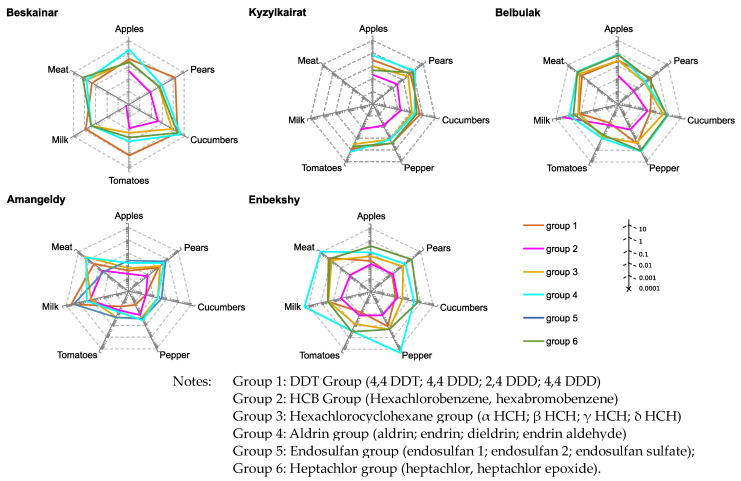
Long-term health risk for populations living in close proximity to the sites of unutilized and obsolete pesticide stocks and consuming food contaminated by POPs.

The input of each evaluated factor to human health and genome stability has been assessed (Figure 3).

It was determined that the overall variability as a result of the combined action of all risk factors in the study sample is 24.74% for health status and 14.24% for genetic status, while the remaining part goes for unaccounted factors. Surprisingly, that age did not reveal any significant influence on health (1%) or mutation rate (0.45%).

The calculation of the predictive ability of the obtained formulas showed that when calculating health risks, the final score predicted the development of chronic diseases in 108 cases (72%), and the presence of chromosomal aberrations in 104 cases (69%). Presumably, these values are associated with the likely inaccuracy of the data obtained during the survey, as well as the possible lack of other important genes in the assessment of genetic systems.

## 4. Discussion

### 4.1. Overview of Pesticide Contamination and Calculation of Risks

The results of studies on human exposure to pesticides suggest that the dose-response relationship, sex, and chemical class of POPs influence their bioaccumulation in adipose tissue, as well as their effector mechanisms and toxicity. However, the mechanisms of these relationships are not clearly defined. For example, it is not clear why some studies indicate a more common association between POP exposure and obesity/diabetes in women than in men or vice versa [51,52]. The relationship between pesticide exposure effects and individual factors such as age, body weight, smoking, and drinking is still unclear, and most related studies give contradictory results [21].

Although short-term and long-term risk assessments give good results for general risks, they are not able to take into account specific factors because they are based on average consumption and pollution. In a more detailed analysis after conducting a survey in the villages, it was found that eating habits are very different for each of them. It depends on the availability of certain products, the level of prosperity of the population, and other factors. This fact, along with individual characteristics, such as gene polymorphisms inherited by people, which give increased susceptibility, or, conversely, resistance to the action of toxicants, as well as factors of age, the presence of bad habits, and individual eating habits, vary significantly within a population and can have a strong influence on risk assessment at the personal level.

Even though our studies have not shown a significant effect of body weight as a risk factor for health and genetic status in the presence of pesticide contamination, it is known that the biochemical characteristics of POPs allow them to accumulate in adipose tissue [47,48,49]. In this regard, the study of an isolated Arctic Inuit population, which mainly feeds on marine food, deserves special attention. Bioaccumulating in fish and marine mammals, the spectrum of organochlorine pesticides enters their food chain. Very high concentrations of a wide range of pesticides were recorded in Inuit adults, including pregnant women, newborns, and fetuses [53,54]. The poor health status of the Inuits may be explained by their isolation, aging, and high pesticide contamination rates. They have an increased risk of developing cardiovascular disease, obesity, diabetes, and hypertension. Furthermore, in this population, a decrease in physical activity and a deterioration in the lipid profile were noted [55].

Bad habits can also increase the toxic effects of pesticides. It was shown that exposure to pesticides increases the risk of arterial hypertension and other cardiovascular diseases in smoking individuals [28,55,56]. Reduced smoking and alcohol consumption, combined with seafood, may reduce pesticide bioaccumulation and the risk of cardiovascular diseases [55]. Alcohol, according to some studies, can also influence the outcome of pesticide poisoning, although this factor remains poorly understood. In a study by Michael Eddleston and coauthors [29], acute organophosphate insecticide dimethoate poisoning was studied in a cohort of patients. A higher risk of death was associated with elevated levels of dimethoate and ethanol in the blood plasma. The authors proposed two possible explanations. One of these is the uncontrolled contact of drinking people with pesticides. Another hypothesis relates to ethanol’s influence on the delay in dimethoate metabolism and detoxication. So, the mechanisms of ethanol interactions with pesticides in the human body should be studied more.

Studies on the effect of age on risks from pesticide exposure also provide conflicting results. Both increased sensitivity of the body to the action of pesticides at a younger age and greater risks of developing the disease in cases of pesticide poisoning in the elderly are noted.

Studies on mice divided into young and old groups showed differences in the absorption, detoxification, and biotransformation of pesticides in the body depending on age [56]. C. Maurice et al., hypothesized that prenatal exposure to organochlorine compounds negatively affects the reproductive function and health of older men. Using an aging rat model, it has been determined that prenatal exposure to organochlorine compounds impairs sperm motility in adult rats at postnatal day 90, which is approximately equivalent to the age of a 30-year-old male, and that older rats are infertile [57].

Other studies of the effects of organochlorine pesticides in mice, using DDT as an example, have shown the possibility of long-term effects of neonatal exposure to DDT [58,59]. In rats aged 10 days, the total density of cholinergic muscarinic receptors increased in the cerebral cortex one week after exposure to DDT, but no effect was noted in the hippocampus. In addition, muscarinic receptor binding was still altered at 4 months of age after a single DDT administration, but a decrease in binding density was observed concurrently. Functional changes (deficit in locomotor habituation) were also noted in rats 4 months after acute exposure to DDT.

Human studies indicate an increased sensitivity in elderly people to the effects of pesticides. Thus, in a study by Liu et al., it was demonstrated that age over 50 years was a risk factor for death in cases of intoxication with organophosphorus compounds [60]. Jia-Ruei Yu’s study followed 71 elderly patients with organophosphate poisoning. An acute cholinergic crisis developed in all patients (100.0%). Complications included respiratory failure (52.1%), aspiration pneumonia (50.7%), acute renal failure (43.7%), severe impairment of consciousness (25.4%), shock (14.1%), and seizures (4.2%). Some patients also developed intermediate syndrome (15.5%) and delayed neuropathy (4.2%) [61].

**Figure 4:**
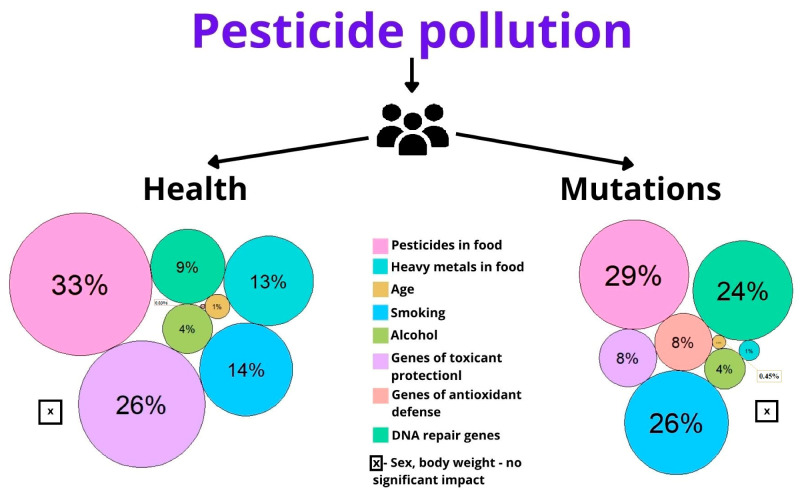
Influence of studied factors on health and genetic status.

In our study, the age factor showed the least degree of influence in assessing the effect of pesticides on health and genetic status (Figure 4). This, as noted earlier, may be due not only to the relative homogeneity of the sample in terms of age but also to the effect of stabilizing selection. In the studied villages, most of the surveyed people lived in this territory for a long time (more than 15 years), very often from birth. We hypothesize that only people whose genetic state conferred some degree of resistance to the effects of pesticide contamination were able to live in these areas for so long, while other people either moved to other settlements or died.

Gender differences in pesticide sensibility as a potential factor were also investigated in some studies. Edward J. Kasner et al., describe higher rates of pesticide poisoning among females than among males [62]. However, the authors suggest that this difference in risks could be due to male and female workers working with different kinds of crops on farms. Our study does not reveal any significant differences in calculated individual risks among males and females who have the same lifestyle risks. For example, there were no differences in risk for a family of three (sample code ПЦ-139—0.685 risk index; ПЦ-140—0.659, ПЦ-142—0.734, correspondingly), living in one house and sharing the same food habits, and even genetics showed no difference for males and females.

In this study, in addition to examining health risks, the possible genotoxic effect of pesticides was also investigated. To study the mutagenic impact of pollution on the population of the region, the cytogenetic method was chosen as the most optimal one.

Assessment of genotoxicity using cytogenetic methods is widely used around the world. Methods for assessing genotoxicity include the micronucleus test, the comet test, and the chromosomal aberration scoring method. An earlier study showed a correlation between the level of chromosome aberrations and DNA repair gene polymorphisms [32]. Based on these data, we included other likely risk factors in the multivariate mutation risk analysis by introducing a scoring system for assessing the action of genes.

The proposed scoring system for assessing the performance of genetic systems makes it possible to calculate the effect of each gene polymorphism in this system and to give an overall assessment of the performance of the entire genetic system for subsequent inclusion in the formula for multifactorial analysis of individual risks. A similar approach was used in a patented formula to calculate cervical cancer risk based on the methylation of risk genes [63].

Our study provides the results of a comprehensive analysis of several risk factors for pesticide exposure, taking into account data that was collected individually. A similar study, in which the authors also used a multiple regression method for the evaluation of DNA damage risks from pesticide exposure, also marked GSTP1 and XRCC1 gene polymorphisms among the main risk factors [64].

The association of pesticide contamination with epigenetic mechanisms is studied very actively [65,66,67]. These studies show that organochlorine pesticides can induce changes in epigenetic patterns that influence metabolic violations. A study by Maurice, C. et al., showed that exposure to OCPs affects sperm DNA methylation in male mice, and the effect is consistent through at least two generations. That could include stillbirths, congenital defects, placental abnormalities, poor fetal growth, and an overall shortened lifespan [65]. A. Lismer et al., also showed that in men, exposure to DDT and p,p′-DDE affects the sperm epigenome in a dose-dependent manner and may negatively affect the health of future generations through epigenetic mechanisms [66].

In the study of pesticide association with DNA methylation [68], the research used a method of linear regression to assess the levels of correlation between pesticide exposure and a number of affected methylation sites.

Although the multiple regression method itself is used in risk assessment in other studies, this paper offers some new approaches to the selection of risk factors.

### 4.2. Genes Impact in Protection of Human Body from Environmental Influence

When we chose the candidate gene variants for inclusion criteria for individual health and genetic risk assessment, we selected genes encoding enzymes participating in key events of organochlorine pesticide biotransformation in the human body. All selected gene polymorphisms strongly associate with hazardous effects on health and can modulate susceptibility to pesticide exposure, as shown in many case-control studies [11,12,13,14,15,16,17,18,19,20,21].

As criteria for the predictive ability of proposed approaches to estimate individual risks of pesticide influence, the existing personal health status and chromosomal aberration level were used.

According to the obtained data, the main impact on individual health risks was recorded for the group of xenobiotic detoxification genes (26%), and the DNA repair system (24%), which was mainly associated with genetic risk expression. It was revealed that the impact of genes in the antioxidant protection system was mostly expressed in genetic risk (8%), but did not significantly influence health risk (0.03%). Therefore, the role of the selected polymorphisms should be discussed more attentively.

There is evidence that mutations in the genes for DNA repair and detoxification of xenobiotics are directly proportional to the number of chromosome aberrations in lymphocytes, and genetic polymorphisms in these genes can affect individual sensitivity to the effects of certain pesticides [19]. *XRCC1*, *XRCC3*, and *XPD* are key genes for DNA repair of chromosomal breaks. Studies show a significant increase in cell damage and cell death for individuals with mutant genotype alleles of the *XRCC1 Arg194Trp* (rs 1799782) gene in comparison with a control group that was not exposed to pesticides [21]. Polymorphisms in the *XRCC1* gene are associated with a decrease in the ability of the protein product of this gene to repair DNA, an increase in the number of gene mutations, and the risk of tumor formation [44,45,67]. Our previous study also showed an increase in the number of chromosome aberrations in individuals with mutant alleles of the *XRCC3* (rs 861539) and *XPD* (rs13181) genes [32].

The study showed a strong influence of polymorphisms in detoxification genes on the health status of the population against the background of persistent pesticide pollution. These data confirm the conclusions of many studies devoted to the problem of assessing genetic factors in pesticide contamination.

There are data indicating the possibility of using polymorphic variants of xenobiotic metabolism genes as molecular genetic markers for assessment of the individual sensitivity of the population to adverse environmental influences [46,47]. Pesticide toxicity, in combination with a poor status in detoxification, can increase the risk of cancer [69,70].

The members of the cytochrome P450 (CYP) gene family are involved in the I stage of xenobiotic and drug metabolism. Many of them are associated with the development of cancer [71] and a number of other diseases, such as arthritis, allergies, allergic dermatitis, miscarriages, etc. [72,73]. There is evidence of the involvement of the *CYP* enzymes in the mechanism of action of the pesticides DDT and DDE in the human placenta [74]. For example, *CYP2D6* is known to decrease its activity in the context of pesticide exposure, raising the risk of neurotoxicity [75,76] and Parkinson’s disease [77]. It was shown that endosulfan-class pesticides can significantly increase *CYP2B6* promoter activity [77]. In rats, organochlorine pesticides (OCPs) such as DDT and methoxychlor strongly induce the transcription of CYP2B isoforms and, to a lesser extent, members of the CYP3A subfamily. OCPs have antiandrogenic effects and can suppress androgen-mediated gene activation [78]. Thus, xenobiotic detoxification genes play a key role in assessing the risks associated with chronic pesticide exposure [79].

The most studied is the polymorphism of the glutathione S-transferase (*GST*) superfamily genes involved in the second phase of xenobiotic detoxification (transport of toxic compounds from the cell). Enzymes of the *GST* family have a high degree of polymorphism, which determines the different individual sensitivity of organisms to the action of mutagenic factors, including pesticide contamination. Genes of the *GST* family (M1, T1, and P1 types) may play the role of predisposing factors in the process of carcinogenesis [69,70]. Boada et al., have shown that the risk of developing bladder cancer against the background of environmental pollution with DDT increases when polymorphisms of the *GSTM1* and *GSTT1* genes are introduced into statistical calculations, while without taking into account these mutations, there is no risk at all [70]. It is reported that exposure to organochlorine pesticides for pregnant women with mutant variants of *GSTT1* and GSTM1 significantly increases the risk of fetal growth restriction [80].

It has been shown that some pesticides have the ability to influence DNA by inducing oxidative stress via direct or indirect means [81]. The key enzymes in antioxidant systems are superoxide dismutase 1 (SOD1) and glutamate cysteine ligase (GCLM and GCLC subunits). Polymorphisms of genes encoding antioxidant enzymes can be associated with susceptibility to the development of multifactorial diseases, such as cardio-vascular diseases, amyotrophic lateral sclerosis [79], Parkinson’s disease, and hemolytic anemia [82]. The transcription factor NFE2L3, which binds antioxidant response elements in target genes, is involved in various cellular processes, including carcinogenesis, stress response, differentiation, and inflammation [48,49,50,83].

Although the polymorphisms of genes responsible for xenobiotic detoxification, DNA repair, and antioxidant defense are intensively studied regarding their health effects, there are still very little data about their interactions with pesticides and the combined organism’s response to chronic toxic exposure. Possibly, further studies will provide new data about the involvement of other genetic systems in pesticide biotransformation in the human body.

We assume that the results of this study can be useful for understanding the mechanisms of health problems induced by organochlorine pesticides.

## 5. Conclusions

Although the results of the study look promising, the reliability of the obtained data is still not sufficient, so further research involving larger samples is needed. The study of gene polymorphisms and their interactions with other risk factors and between each other could reveal new data and give a better understanding of the mutagenic effects of pesticides and the mechanisms of pathogenesis of diseases caused by pesticide exposure, as well as help develop better methods of risk evaluation.

Thus, our proposed method for assessing individual risk for people chronically exposed to pesticides takes into account both individual characteristics (age, bad habits, etc.), including individual consumption (individual pesticide intake), and genetic status for the genes involved in the metabolism of pesticides for each individual. Perhaps this approach can be more informative than the general population risk assessment and will help develop more effective measures for the prevention and treatment of diseases associated with the action of POPs.

## Figures and Tables

**Table 1:** Unique primers, amplification and restriction conditions, and target products for determining the genotype by studied gene polymorphisms.

Gene	Primers	PCR Conditions	Restriction Endonuclease	Restriction Products (b.p.)
*GSTP1* Ile^105^Val	(F) 5′-ACC CCA GGG CTC TAT GGG AA-3′ (R) 5′-TGA GGG CAC AAG AAG CCC CT-3′	30 cycles: 94 °C—30 s 55 °C—30 s 72 °C—30 s	Alw26I	Val/Val: 91 + 85; Ile/Val: 176 + 91 + 85; Ile/Ile: 176.
*XRCC1* Arg^399^Gln	(F) 5′-CAA GTA CAG CCA GGT CCT AG-3′ (R) 5′-CCT TCC CTC ATC TGG AGT AC-3′	40 cycles: 94 °C—15 s 55 °C—30 s 72 °C—45 s	NciI	Arg/Arg: 89 + 59 Arg/Gln: 248 + 159 + 89 Gln/Gln: 248
*XRCC1* Arg^194^Trp	(F) 5′-GCC CCG TCC CAG GTA-3′ (R) 5′-AGC CCC AAG ACC CTT T-3′	40 cycles: 94 °C—15 s 57 °C—45 s 72 °C—45 s	PvuII	Arg/Arg: 490 Arg/Trp: 490 + 294 + 196 Trp/Trp: 294 + 196
*XRCC3* Met^241^Trp	(F) 5′-GCC TGG TGG TCA TCG ACT C-3′ (R) 5′-ACA GGG CTC TGG AAG GCA CTG CTC AGC TCA CGC ACC-3′	40 cycles: 94 °C—15 s 60 °C—30 s 72 °C—45 s	Nco1	Trp/Trp: 136 Trp/Met: 136 + 97 + 39 Met/Met: 97 + 39
*XPD* Lys751Gln	(F) 5′-GCC CGC TCT GGA TTA TAC G-3′ (R) 5′-CTA TCA TCT CCT GGC CCC C-3′	38 cycles: 94 °C—45 s 60 °C—45 s 72 °C—60 s	PstI	Lys/Lys: 290 + 146 Gln/Gln: 227 + 146 + 63 Lys/Gln: 290 + 227 + 146+ 63
*GSTT1*	(F) 5′-CCT TAC TGG TCC TCA CAT CTC-3′ (R) 5′-TCA CCG GAT CAT GGC CAG CA-3′	35 cycles: 94 °C—2 min 59 °C—1 min 72 °C—1 min	_	+/+;+/−: 480
*GSTM1*	(F) 5′-GAA CTC CCT GAA AAG CTA AAG C-3′ (R) 5′-GTT GGG CTC AAA TAT ACG GTG G-3′			+/+;+/−: 215
*β*-globin	(F) 5′-CAA CTT CAT CCA CGT TCA CC-3′ (R) 5′-GAA GAG CCT AGG ACA GGT AC-3′			+/+: 268
*GPX4*	(F) 5′-GAG AAG GAC CTG CCC CAC TA-3′ (R) 5′-GTC ATG AGT GCC GGT GGA AG-3′	35 cycles: 95 °C—30 s, 61 °C—30 s, 72 °C—45 s,	StyI	TT: 68 + 28; TC: 96 + 68 + 28; CC: 96.
*GCLC*	(F) 5′-TCG TCC CAA GTC TCA CAG TC-3′ (R) 5′-CGC CCT CCC CGC TGC TCC TC-3′	35 cycles: 95 °C—30 s, 61 °C—30 s, 72 °C—45 s,	Tsp45I	CC: 500 + 113; CT: 500 + 302 + 198 + 113; TT: 302 + 198 + 113.
*GCLM*	(F) 5′-CTC AAG GGC AAA GAC TCA-3′ (R) 5′-CCG CCT GGT GAG GTA GAC AC-3′	35 cycles: 95 °C—30 s, 58 °C—30 s, 72 °C—45 s,	MspI	CC: 200 + 84 + 45; CT: 200 + 129 + 84 + 45; TT: 200 + 129.

**Table 2:** Main characteristics of cohorts exposed by pesticides pollution.

Locality	Total Persons	Ethnicity, pers. (%)	Males (%)	Females (%)	Years of Birth (Average Age)
Kyzylkairat	32	Kazakhs—27 (84%) Russians—1 (3%) Others—4 (13%)	10 (31%)	22 (69%)	1947–1999 (48.6 ± 12.8)
Belbulak	26	Kazakhs—14 (54%) Russians—7 (27%) Others—5 (19%)	5 (19%)	21 (81%)	1942–2004 (49.5 ± 13.6)
Beskainar	31	Kazakhs—25 (81%) Russians—5 (16%) Others—1 (3%)	7 (23%)	24 (77%)	1950–1989 (52.8 ± 11.8)
Amangeldy	25	Kazakhs—18 (72%) Russians—4 (16%) Others—3 (12%)	14 (56%)	11 (44%)	1948–1997 (50.6 ± 12.8)
Enbekshi	27	Kazakhs—21 (78%) Russians—6 (22%)	8 (30%)	19 (70%)	1943–1996 (50.6 ± 13.3)
Karakastek	25	Kazakhs—24 (96%) Russians—1 (4%)	4 (16%)	21 (84%)	1939–1997 (50.48 ± 3.14)
Umbetaly	25	Kazakhs—25 (100%)	10 (40%)	15 (60%)	1954–1999 (40.84 ± 2.91)

**Table 3:** Mean levels of products consumption per day (kg; L) for each cohort.

Locality	Meat	Cucumbers	Tomato	Pepper	Apples	Pears	Milk
Kyzylkairat	0.14 ± 0.12	0.13 ± 0.16	0.18 ± 0.21	0.07 ± 0.087	0.28 ± 0.33	0.11 ± 0.14	0.65 ± 0.60
Belbulak	0.14 ± 0.09	0.07 ± 0.06	0.11 ± 0.09	0.03 ± 0.02	0.40 ± 0.30	0.15 ± 0.17	0.35 ± 0.29
Beskainar	0.14 ± 0.11	0.21 ± 0.17	0.46 ± 0.35	0.12 ± 0.14	0.60 ± 0.50	0.67 ± 0.62	0.41 ± 0.37
Amangeldy	0.16 ± 0.11	0.32 ± 0.17	0.58 ± 0.29	0.12 ± 0.12	0.62 ± 0.41	0.79 ± 0.58	0.93 ± 0.43
Enbekshi	0.05 ± 0.03	0.19 ± 0.14	0.35 ± 0.21	0.02 ± 0.03	0.10 ± 0.12	0.10 ± 0.13	0.51 ± 0.54
Karakastek	0.32 ± 0.3	0.26 ± 0.21	0.46 ± 0.33	0.22 ± 0.19	0.38 ± 0.38	0.13 ± 0.16	0.40 ± 0.26
Umbetaly	0.19 ± 0.13	0.47 ± 0.36	0.53 ± 0.35	0.19 ± 0.30	0.42 ± 0.43	0.31 ± 0.39	0.53 ± 0.39

**Table 4:** Mean body weight in surveyed cohorts.

	Karakastek	Belbulak	Beskainar	Amangeldy	Enbekshi	Karakastek	Umbetaly
**Mean weight for males, kg**	71.9 ± 9.17	79.1 ± 12.13	79.83 ± 11.21	68.09 ± 5.36	76 ± 2.67	78.67 ± 14.22	70.7 ± 11.9
**Mean weight for females, kg**	68.54 ± 9.1	73.76 ± 14.72	70.37 ± 10.78	68.45 ± 13.42	71.38 ± 10.13	69.09 ± 9.1	64.93 ± 7.67
***t*-test**	0.0216						

**Table 5:** Results of study cohort genotyping on key gene variants involved in pesticide biotransformation.

Name	Gene	Number of Individuals with Recorded Genotypes			Allele Frequencies		χ^2^ *p* Value
		AA	AB	BB	A	B	
rs861539	*XRCC3*	95	38	18	0.755	0.245	15.445 0.0004
rs1799782	*XRCC1*	102	41	8	0.811	0.189	1.940 0.379
rs25487	*XRCC1*	50	63	38	0.540	0.460	3.878 0.144
rs13181	*XPD*	88	55	8	0.765	0.235	0.025 0.988
rs138002121	*SOD1*	133	1	17	0.884	0.116	141.398 0
rs1041740	*SOD1*	59	55	37	0.573	0.427	9.875 0.007
rs17861084	*CYP1A1*	134	0	17	0.887	0.113	151.000 0
rs8192718	*CYP2B6*	132	2	17	0.881	0.119	132.532 0
rs186133763	*CYP2D6*	134	0	17	0.887	0.113	151.000 0
rs11592737	*CYP2C19*	115	29	7	0.858	0.142	6.890 0.032
Deletion	*GSTT1*	128 (+/+, +/−)		89 (−/−)	0.360 (+)	0.640 (−)	
Deletion	*GSTM1*	110 (+/+, +/−)		110 (−/−)	0.500 (+)	0.500 (−)	
rs1138272	*GSTP1*	115	17	19	0.818	0.182	58.435 0
rs1695	*GSTP1*	87	50	14	0.742	0.258	2.783 0.249
rs1871042	*GSTP1*	72	48	31	0.636	0.364	14.854 0.0006
rs2237329	*NFE2L3*	106	23	22	0.778	0.222	47.158 0
rs713041	*GPX4*	21	97	33	0.460	0.540	12.957 0.002
rs41303970	*GCLM*	117	27	7	0.864	0.136	8.554 0.014
rs12524550	*GCLC*	131	3	17	0.877	0.123	124.384 0
rs3799694	*GCLC*	81	55	15	0.718	0.282	1.494 0.474
rs524553	*GCLC*	126	23	2	0.911	0.089	0.628 0.730

Notes: A—ancestral allele; B—polymorphic or mutant allele.

**Table 6:** Estimated mean exposure of studied population to different pesticide groups (mg/kg *Bw* per day), based on chemical analysis of products, with ADI for each group according to WHO.

Villages	DDT	HCB	HCH	Aldrin	Endosulfans	Heptachlor
Kyzylkairat	0.40 ± 0.7	0.008 ± 0.01	0.03 ± 0.05	0.07 ± 0.11	0.14 ± 0.21	0.04 ± 0.07
Belbulak	0.18 ± 0.3	0.04 ± 0.08	0.06 ± 0.1	0.094 ± 0.16	0.14 ± 0.20	0.07 ± 0.10
Beskainar	1.26 ± 2.16	0.12 ± 0.22	0.05 ± 0.08	0.27 ± 0.49	0.8 ± 1.32	0.07 ± 0.12
Amangeldy	0.26 ± 0.49	0.007 ± 0.01	0.02 ± 0.04	0.14 ± 0.26	0.08 ± 0.14	0.05 ± 0.10
Enbekshi	0.21 ± 0.36	0.01 ± 0.02	0.06 ± 0.09	0.2 ± 0.34	0.1 ± 0.125	0.09 ± 0.12
Karakastek	0.002 ± 0.003	0.001 ± 0.001	<0.0001	0.0001	<0.0001	<0.0001
Umbetaly	0.002 ± 0.002	0.001 ± 0.001	<0.0001	0.0001	<0.0001	0.001 ± 0.001
ADI	0.01	-	-	0.0002	0.006	0.0001

**Table 7:** Estimated mean exposure of studied population to different pesticide groups via food chain (mg/kg *Bw* per day), taking into account individual food habits, with ADI for each group according to WHO.

Villages	DDT	HCB	HCH	Aldrin	Endosulfans	Heptachlor
**Kyzylkairat**	0.11 ± 0.1	0.04 ± 0.03	0.02 ± 0.012	0.05 ± 0.04	0.08 ± 0.06	0.003 ± 0.003
**Belbulak**	0.04 ± 0.03	0.03 ± 0.02	0.02 ± 0.007	0.04 ± 0.03	0.03 ± 0.01	0.02 ± 0.009
**Beskainar**	0.56 ± 0.3	0.02 ± 0.01	0.02 ± 0.009	0.04 ± 0.02	0.36 ± 0.24	0.01 ± 0.006
**Amangeldy**	0.44 ± 0.2	0.004 ± 0.002	0.011 ± 0.005	0.02 ± 0.006	0.03 ± 0.01	0.01 ± 0.005
**Enbekshi**	0.03 ± 0.03	0.001 ± 0.001	0.005 ± 0.004	0.006 ± 0.005	0.02 ± 0.01	0.006 ± 0.005
**Karakastek**	0.0002 ± 0.0001	0.0001 ± 0	<0.0001	0.0001	<0.0001	<0.0001
**Umbetaly**	0.0002 ± 0.0001	0.0001 ± 0	<0.0001	0.0001	<0.0001	<0.0001
**ADI**	0.01	-	-	0.0002	0.006	0.0001

## Data Availability

The data presented in this study are available on request from the corresponding author. The data are not publicly available due to privacy protection of people who participated in this study.

## Supplementary Materials

The following supporting information can be downloaded at: https://www.mdpi.com/article/10.3390/toxics11060482/s1. Figure S1: Questionaries used for survey of people; Table S1: Full data of the survey in villages of Talgar and Dzhambyl regions; Table S2: MPC data for pesticides in European Union and Customs Union; Figure S2: An example of primary data processing using Illumina GenomeStudio v.2.05 software; Figure S3: An example of genotyping and annotation data processing; Table S3: Characteristics of functions of genes selected for study of pesticides effects on health.
