# Supplementary material for: Individual Risk Assessment for Population Living on the Territories Long-Term Polluted by Organochlorine Pesticides
Source: Toxics. 2023 May 25;11(6):482. doi: 10.3390/toxics11060482 (PMC10304191; doi:10.3390/toxics11060482)
Supplement: Supplementary file 1 [file toxics-11-00482-s001.zip › toxics-2385883-SI.pdf]

Supplementary Materials

Figure S1: Questionnaires used for survey of people.

Name

____

Sex

☐ male
☐ female

Age

____

How long have you been living in this village?

☐ less than 1 year
☐ 1-5 years
☐ 6-10 years
☐ более 10 лет

How many times a day do you eat?

	Several times a day	Once a day	Few times a week	Less often	Don't eat at all
Meat					
Cucumbers					
Tomatoes					
Bell pepper					
Apples					
Pears					
Milk					
Honey					

What size is your usual portion of meat (in grams)?

☐ 20-30 g.
☐ 50 g.
☐ 100-120 g.
☐ 300-400 g.
☐ 500 g. and more

How many cucumbers do you eat at a time / use for cooking?

☐ 1 piece
☐ 2 pieces
☐ 3 pieces
☐ 4 pieces
☐ 5 and more

How many tomatoes do you eat at a time / use for cooking?

☐ 1 piece
☐ 2 pieces
☐ 3 pieces
☐ 4 pieces
☐ 5 and more

What seasons are more typical for your products consumption:

	Winter	Spring	Summer	Autumn
Meat				
Cucumbers				
Tomatoes				
Bell pepper				
Apples				
Pears				
Milk				
Honey				

Please indicate how much food (in grams/liters) do you purchase on average:

1. Meat:
2. Cucumbers:______________________________
3. Tomatoes:
4. Bell pepper:
5. Apples:______________________________
6. Pears:
7. Milk:______________________________
8. Honey

How do you process fruits and vegetables before eating?

☐ Don't wash and clean
☐ Wash but don't clean
☐ Clean but don't wash
☐ Wash and clean

How many bell peppers do you eat at a time / use for cooking?

☐ 1 piece
☐ 2 pieces
☐ 3 pieces
☐ 4 pieces
☐ 5 and more

How many apples do you eat at a time / use for cooking?

☐ 1 piece
☐ 2 pieces
☐ 3 pieces
☐ 4 pieces
☐ 5 and more

How many pears do you eat at a time / use for cooking?

☐ 1 piece
☐ 2 pieces
☐ 3 pieces
☐ 4 pieces
☐ 5 and more

How much milk do you consume at a time / use for cooking?

- ☐ 1 glass
- ☐ 2 glasses
- ☐ 3 glasses
- ☐ 4 glasses
- ☐ 5 and more

How much honey do you consume at a time / use for cooking?

- ☐ 1 tea spoon
- ☐ 2 tea spoons
- ☐ 3 tea spoons
- ☐ 4 tea spoons
- ☐ 5 and more

How far is the source of foodstuffs from home?:

	Own plot / farm	Neighboring plot/farm	Mobile stall	Shop near home	Shop close to home	Shop far from home	Products are brought from another locality
Meat							
Cucumbers							
Tomatoes							
Bell pepper							
Apples							
Pears							
Milk							
Honey							

Questions, wishes or comments about the quality of the products you consume and notes about health issues:

____

____

____

____

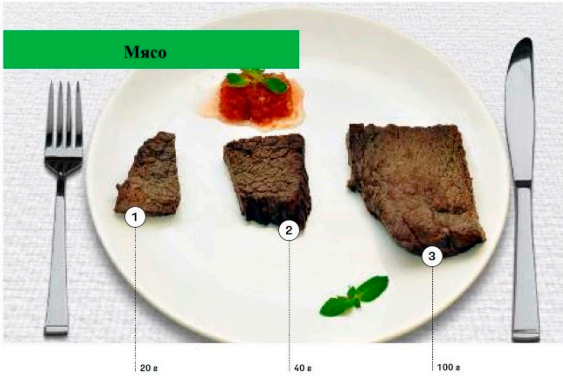

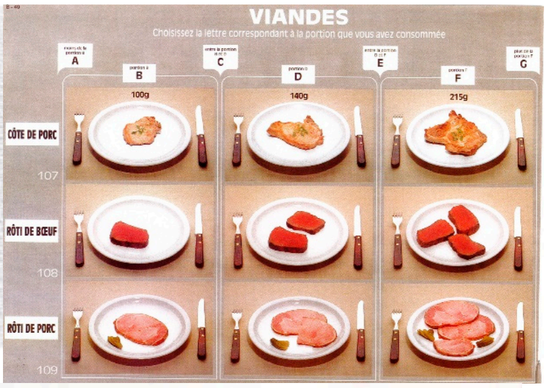

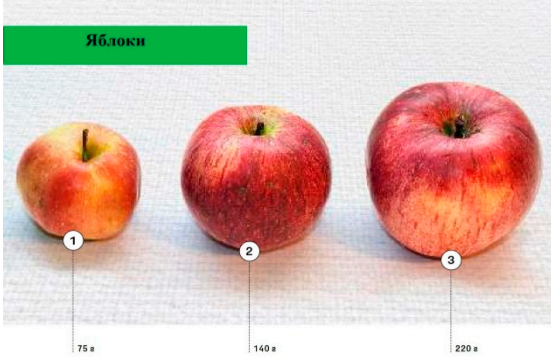

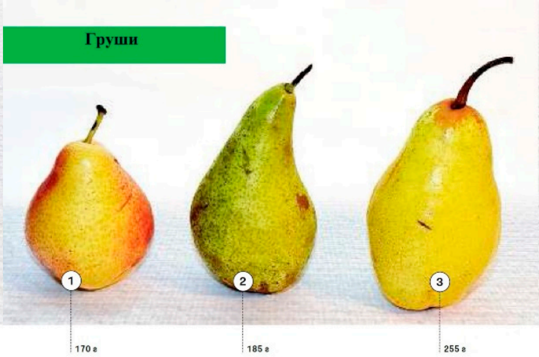

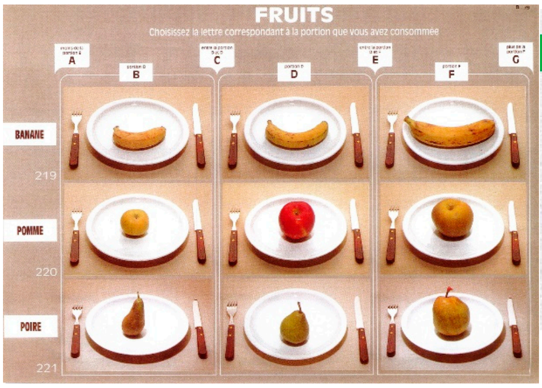

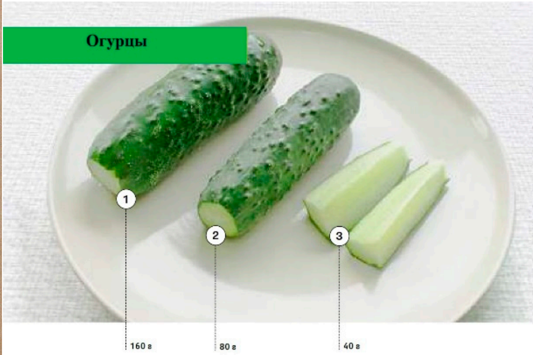

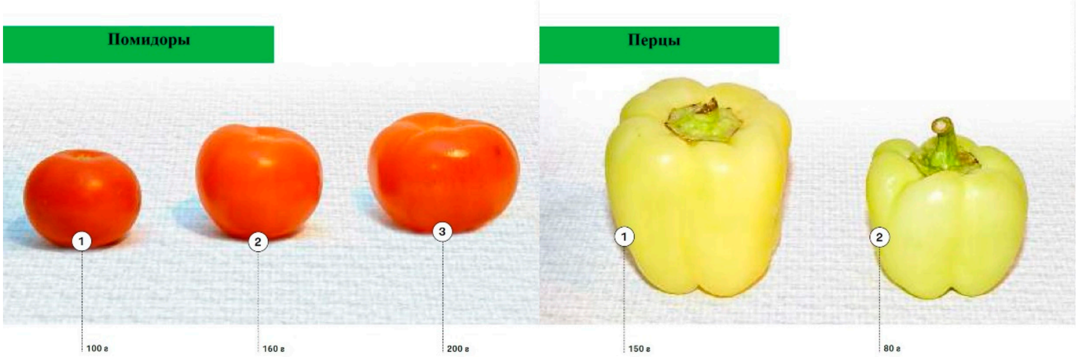

Table S1: Full data of the survey in villages of Talgar and Dzhambyl regions.

**Village**	**Code**	**Year of birth**	**Age**	**Sex**	**Body weight, kg**	**Excess in food**	**Pesticide Excess**	**Excess for heavy metals**	**Reparation system assessment**	**Detoxification system assessment**	**Antioxidant protection system assessment**	**Smoke**	**Drink**
KK	ПЦ-31	1974	46	male	76,2	1,14	0,274	0,067	1	0	0	0	1
KK	ПЦ-32	1984	36	female	71,5	1,00	0,117	0,094	0	0	1	0	0
KK	ПЦ-35	1987	33	male	71,8	3,29	0,854	3,486	0	1	1	1	0
KK	ПЦ-36	1960	60	male	77,5	1,86	0,464	0,770	0	1	0	1	0
KK	ПЦ-37	1987	33	female	72,9	2,43	0,830	0,781	0	2	0	0	0
KK	ПЦ-38	1988	32	male	70	0,71	0,259	0,075	0	2	0	0	0
KK	ПЦ-39	1958	62	female	64	3,29	1,164	3,621	1	1	0	0	1
KK	ПЦ-40	1967	53	female	51,6	1,57	0,092	0,166	0	0	0	0	0
KK	ПЦ-41	1955	65	female	64,4	1,71	0,226	1,158	0	1	0	0	0
KK	ПЦ-42	1986	34	female	60	1,71	0,127	0,638	1	1	0	0	0
KK	ПЦ-43	1991	29	female	62,4	1,29	0,076	0,138	1	2	0	0	0
KK	ПЦ-44	1947	73	male	50,2	2,29	0,374	0,291	0	1	0	1	1
KK	ПЦ-46	1948	72	female	73,3	0,43	0,125	0,044	0	2	0	0	1

KK	ПЦ-47	1963	57	female	54,4	1,14	0,266	0,238	1	1	0	0	0
KK	ПЦ-48	1991	29	female	60	3,00	0,380	2,018	1	1	0	0	0
KK	ПЦ-50	1957	63	female	80	0,43	0,102	0,055	0	1	1	0	0
KK	ПЦ-51	1955	65	female	71,4	3,00	0,578	1,223	0	0	0	0	0
KK	ПЦ-52	1955	65	male	94,1	0,43	0,087	0,047	0	0	1	0	0
KK	ПЦ-53	1964	56	female	93,6	1,29	0,157	0,187	0	3	1	0	0
KK	ПЦ-54	1951	69	female	60	1,71	0,115	0,429	0	0	1	0	1
KK	ПЦ-57	1972	48	female	62,7	2,14	0,135	0,505	0	1	0	0	1
KK	ПЦ-59	1947	73	female	80,6	1,57	0,091	0,204	0	1	1	0	0
KK	ПЦ-60	1969	51	male	63,5	1,71	0,115	0,258	0	1	1	1	0
KK	ПЦ-62	1980	40	female	82,4	1,57	0,089	0,223	0	2	1	0	0
BB	ПЦ-94	1958	62	male	97,3	1,86	0,266	1,065	0	0	0	0	0
BB	ПЦ-96	1942	78	female	67,3	2,00	0,095	1,273	2	2	0	0	0
BB	ПЦ-97	1952	68	female	60	1,14	0,111	0,491	0	2	0	0	0
BB	ПЦ-98	1966	54	female	60	2,29	0,344	1,948	1	1	1	0	0
BB	ПЦ-99	1972	48	female	68,3	1,71	0,121	0,812	0	1	0	0	0
BB	ПЦ-100	1963	57	female	81,3	2,14	0,088	1,314	0	3	1	0	0
BB	ПЦ-101	1957	63	female	60	1,43	0,097	0,494	1	1	1	0	0
BB	ПЦ-102	1986	34	female	95,1	1,57	0,082	0,486	2	1	0	0	0
BB	ПЦ-104	2004	16	male	70	1,71	0,087	0,489	1	0	0	0	0
BB	ПЦ-106	1970	50	female	76	1,43	0,164	0,507	1	1	0	0	0

BB	ПЦ-107	1960	60	female	65,3	2,00	0,122	0,641	1	0	1	0	0
BB	ПЦ-108	1949	71	female	89,2	1,57	0,072	0,423	1	1	0	0	0
BB	ПЦ-111	1990	30	female	42,4	2,29	0,484	2,756	0	1	0	0	0
BB	ПЦ-112	2003	17	male	70	2,00	0,103	0,961	1	1	0	0	0
BB	ПЦ-113	1963	57	female	57,9	2,29	0,354	2,019	0	1	0	0	0
BB	ПЦ-114	1981	39	female	146,9	2,00	0,045	0,269	0	2	0	0	0
BB	ПЦ-115	1965	55	female	76,6	1,29	0,095	0,976	2	1	0	0	0
BB	ПЦ-116	1975	45	female	59,8	2,14	0,140	1,999	1	2	0	0	0
BB	ПЦ-117	1973	47	female	70,5	2,14	0,267	1,190	0	1	0	0	0
BB	ПЦ-118	1971	49	female	83,7	2,14	0,131	1,264	0	1	0	0	1
BB	ПЦ-119	1994	26	female	67,4	2,29	0,279	1,245	1	2	0	0	0
BK	ПЦ-63	1957	63	female	66	2,86	1,733	1,944	0	1	0	1	0
BK	ПЦ-64	1959	61	female	79,3	2,71	1,110	0,996	1	0	1	0	0
BK	ПЦ-65	1960	60	female	74,3	2,86	0,466	0,417	0	1	0	0	0
BK	ПЦ-66	1961	59	male	94,8	2,86	1,636	1,676	1	1	0	0	1
BK	ПЦ-67	1956	64	male	98,5	2,57	0,367	0,317	0	1	0	1	1
BK	ПЦ-68	1968	52	male	72,2	3,29	1,440	1,424	0	3	0	0	0
BK	ПЦ-69	1968	52	female	60	3,29	1,657	1,699	1	1	0	0	0
BK	ПЦ-70	1971	49	female	60	2,57	1,051	1,093	0	2	0	0	0
BK	ПЦ-71	1969	51	female	99	1,43	0,190	0,199	0	2	0	0	0
BK	ПЦ-74	1951	69	male	59	3,29	1,367	1,309	0	1	0	1	0

BK	ПЦ-75	1964	56	female	92,1	1,71	0,125	0,083	0	1	1	0	0
BK	ПЦ-76	1957	63	female	59,3	3,00	1,129	1,173	0	1	1	0	0
BK	ПЦ-80	1963	57	female	53,1	1,71	0,322	0,078	0	1	1	0	0
BK	ПЦ-81	1984	36	female	70,8	2,14	0,444	0,333	0	2	0	0	0
BK	ПЦ-82	1962	58	female	100,7	2,71	1,934	2,171	2	2	1	0	1
BK	ПЦ-83	1966	54	male	77,1	1,71	0,193	0,125	0	2	0	0	0
BK	ПЦ-84	1980	40	male	77,4	3,00	1,278	1,258	0	2	0	1	0
BK	ПЦ-86	1959	61	female	60	2,14	0,998	1,228	0	2	1	0	0
BK	ПЦ-87	1950	70	female	60	2,71	1,985	1,922	0	1	0	0	0
BK	ПЦ-88	1977	43	female	69,3	2,14	0,516	0,346	0	1	1	0	0
BK	ПЦ-89	1970	50	female	60	3,14	1,926	1,436	0	2	0	0	0
BK	ПЦ-90	1966	54	female	60	2,00	0,159	0,072	1	1	0	0	0
BK	ПЦ-91	1968	52	female	69,3	2,71	1,719	1,664	0	2	1	0	1
BK	ПЦ-93	1987	33	female	73,4	1,71	0,279	0,200	1	1	0	0	0
AG	ПЦ-120	1956	64	male	61	1,14	0,620	0,479	1	0	1	1	0
AG	ПЦ-122	1959	61	male	63	1,86	0,307	2,510	1	2	2	1	1
AG	ПЦ-123	1976	44	female	59	1,86	1,059	4,841	1	1	0	0	1
AG	ПЦ-124	1978	42	male	79	1,14	0,356	1,888	0	1	0	1	1
AG	ПЦ-126	1970	50	male	76	1,86	0,255	2,080	0	1	1	0	0
AG	ПЦ-127	1959	61	female	125	1,57	0,155	2,294	1	1	0	0	1
AG	ПЦ-128	1948	72	male	55	2,29	1,371	7,343	0	2	1	0	0

AG	ПЦ-129	1997	23	male	71	1,14	0,216	2,475	0	0	1	0	0
AG	ПЦ-130	1965	55	female	65	2,14	0,642	4,547	1	0	0	0	0
AG	ПЦ-131	1968	52	male	70	1,57	0,258	2,981	0	0	0	1	1
AG	ПЦ-132	1956	64	male	64	1,57	0,439	2,418	0	0	0	0	1
AG	ПЦ-133	1974	46	female	59	1,00	1,016	2,002	2	1	0	0	1
AG	ПЦ-134	1984	36	female	63,4	1,29	0,220	1,314	2	0	0	0	0
AG	ПЦ-136	1952	68	female	53,1	2,00	0,588	5,004	1	1	0	0	0
AG	ПЦ-137	1988	32	female	67	1,29	0,362	0,770	0	0	0	0	0
AG	ПЦ-138	1985	35	male	72	1,86	0,406	2,242	0	1	0	1	1
AG	ПЦ-139	1973	47	female	79	1,57	0,512	2,284	1	1	0	0	0
AG	ПЦ-140	1971	49	male	68	1,57	0,595	2,654	1	1	0	0	0
AG	ПЦ-142	1995	25	male	70	1,57	0,578	2,578	1	2	0	0	0
AG	ПЦ-143	1958	62	female	56	1,43	0,332	3,612	0	2	0	0	0
AG	ПЦ-144	1958	62	female	58	1,29	0,413	0,842	0	1	0	0	0
EB	ПЦ-173	1956	65	female	90	1,00	0,017	0,212	1	0	0	0	0
EB	ПЦ-174	1943	78	female	56	1,57	0,120	0,253	2	0	1	0	0
EB	ПЦ-176	1964	57	female	88	2,29	0,085	1,024	2	0	0	0	0
EB	ПЦ-177	1975	46	female	67	0,57	0,009	0,326	2	0	0	0	0
EB	ПЦ-179	1981	40	female	76	1,43	0,033	0,999	1	1	0	0	0
EB	ПЦ-182	1998	23	male	75	1,14	0,021	0,254	2	1	1	1	1
EB	ПЦ-183	1991	30	male	73	1,14	0,021	0,261	1	2	1	1	0

EB	ПЦ-184	1957	64	female	67	2,57	0,158	1,453	2	2	2	0	0
EB	ПЦ-186	1986	35	female	72	1,86	0,153	1,587	2	0	1	0	0
EB	ПЦ-187	1954	67	male	80	1,00	0,019	0,238	1	1	0	1	0
EB	ПЦ-190	1978	43	female	55	2,29	0,101	1,097	2	1	1	1	0
Karakestek	УЖ-1	1962	59	female	63	0,00	0,001	0,136	0	0	0	0	1
Karakestek	УЖ-2	1947	74	female	82	0,00	0,000	0,006	0	1	0	1	1
Karakestek	УЖ-3	1977	44	female	73	0,00	0,000	0,082	0	1	0	0	1
Karakestek	УЖ-4	1961	60	female	85	0,00	0,000	0,054	0	0	0	0	0
Karakestek	УЖ-5	1982	39	female	60	0,00	0,000	0,082	0	2	0	0	1
Karakestek	УЖ-6	1964	57	female	59	0,00	0,000	0,059	0	1	1	0	0
Karakestek	УЖ-7	1963	58	female	75	0,00	0,001	0,098	0	0	1	0	0
Karakestek	УЖ-8	1981	40	male	100	0,00	0,000	0,132	1	0	0	1	0
Karakestek	УЖ-9	1939	82	female	60	0,00	0,000	0,042	0	1	0	0	0
Karakestek	УЖ-10	1997	24	female	48	0,00	0,001	0,344	0	1	0	0	0
Karakestek	УЖ-11	1990	31	female	60	0,00	0,001	0,287	1	1	0	0	0
Karakestek	УЖ-12	1984	37	female	73	0,00	0,000	0,021	0	1	0	0	0
Karakestek	УЖ-13	1994	27	female	54	0,00	0,000	0,052	0	1	0	0	0

Karakestek	УЖ-14	1990	31	male	63	0,00	0,000	0,069	0	1	1	1	0
Karakestek	УЖ-15	1948	73	female	60	0,00	0,000	0,130	0	0	0	0	0
Karakestek	УЖ-16	1947	74	female	95	0,00	0,000	0,072	0	1	0	0	1
Karakestek	УЖ-17	1958	63	female	80	0,00	0,001	0,182	0	2	1	0	0
Karakestek	УЖ-18	1953	68	male	73	0,00	0,001	0,209	0	1	0	0	0
Karakestek	УЖ-19	1974	47	female	68	0,00	0,000	0,108	1	2	0	0	0
Karakestek	УЖ-20	1968	53	female	70	0,00	0,001	0,272	0	2	0	0	1
Karakestek	УЖ-21	1970	51	female	63	0,00	0,000	0,107	0	2	0	0	1
Karakestek	УЖ-22	1971	50	female	68	0,00	0,000	0,058	1	1	0	0	1
Karakestek	УЖ-23	1966	55	female	75	0,00	0,000	0,137	0	2	0	0	1
Karakestek	УЖ-24	1964	57	female	83	0,00	0,001	0,257	0	0	1	0	1
Karakestek	УЖ-25	1978	43	female	66	0,00	0,000	0,065	1	2	0	0	1
Umbetaly	УЖ-26	1952	69	male	62	0,00	0,000	0,104	0	1	0	1	0
Umbetaly	УЖ-27	1964	57	male	72	0,00	0,000	0,129	1	0	1	1	0
Umbetaly	УЖ-28	1979	42	female	80	0,00	0,000	0,137	0	1	0	1	1
Umbetaly	УЖ-29	1999	22	female	47	0,00	0,001	0,395	0	0	0	0	0

Umbetaly	УЖ-30	1976	45	female	57	0,00	0,000	0,375	0	0	0	0	0
Umbetaly	УЖ-31	1997	24	male	56	0,00	0,000	0,115	0	2	0	1	0
Umbetaly	УЖ-32	1975	46	male	83	0,00	0,000	0,058	0	2	0	0	0
Umbetaly	УЖ-33	1980	41	female	68	0,00	0,000	0,148	1	1	0	0	0
Umbetaly	УЖ-34	1999	22	female	61	0,00	0,000	0,126	0	1	0	0	0
Umbetaly	УЖ-35	1957	64	male	82	0,00	0,000	0,244	1	2	0	0	0
Umbetaly	УЖ-36	1961	60	female	67	0,00	0,000	0,071	0	1	0	0	1
Umbetaly	УЖ-37	1973	48	female	70	0,00	0,000	0,191	0	1	0	0	0
Umbetaly	УЖ-38	1974	47	male	86	0,00	0,001	0,377	0	0	0	1	0
Umbetaly	УЖ-39	1973	48	female	87	0,00	0,001	0,255	1	1	0	0	0
Umbetaly	УЖ-40	1990	31	male	90	0,00	0,000	0,044	0	0	0	1	0
Umbetaly	УЖ-41	1990	31	male	64	0,00	0,001	0,258	0	0	1	1	1
Umbetaly	УЖ-42	1993	28	male	54	0,00	0,000	0,158	0	0	0	1	1
Umbetaly	УЖ-43	1991	30	female	63	0,00	0,000	0,117	1	2	0	0	0
Umbetaly	УЖ-44	1996	25	female	70	0,00	0,000	0,094	0	1	1	0	0
Umbetaly	УЖ-45	1988	33	female	70	0,00	0,000	0,117	0	1	0	0	0
Umbetaly	УЖ-46	1983	38	female	51	0,00	0,000	0,060	0	1	0	0	0
Umbetaly	УЖ-47	1994	27	female	58	0,00	0,000	0,330	0	1	0	0	0
Umbetaly	УЖ-48	1990	31	female	60	0,00	0,000	0,077	0	1	0	0	0
Umbetaly	УЖ-49	1957	64	male	58	0,00	0,000	0,193	0	1	0	1	0
Umbetaly	УЖ-50	1985	36	female	65	0,00	0,000	0,217	0	2	0	0	0

Table S2: MPC data for pesticides in European Union and Customs Union.

**Pesticide classes**	**MPC standards of the European Union (mg/kg)**	**MPC standards of the Customs Union (mg/kg)**
HCB	0,01	no data
α HCH	0,01	0,05
γ HCH	0,01	0,05
β HCH	0,01	0,05
Heptachlor	0,01	no data
δ HCH	0,01	0,05
Aldrin	0,01	no data
Keltan	0,02	0,01
Heptachlor epoxide	0,01	no data
Chlordane	0,01	0,02
Endosulfan 1	0,05	no data
DDE	0,05	0,01
Dieldrin	0,01	no data
2.4-DDD	0,05	no data
Chlorobenzylate	0,02	no data
DDD	0,01	0,01
Endrin	0,01	0,05
Endosulfan 2	0,05	no data
DDT	0,05	0,01
Endrin aldehyde	0,01	no data
Endosulfan sulfate	0,05	no data
Dibutilendan	no data	no data
Metoxychlor	0,01	no data
Hexabromobenzene	0,01	no data

Figure S2: An example of primary data processing using Illumina GenomeStudio v.2.05 software.

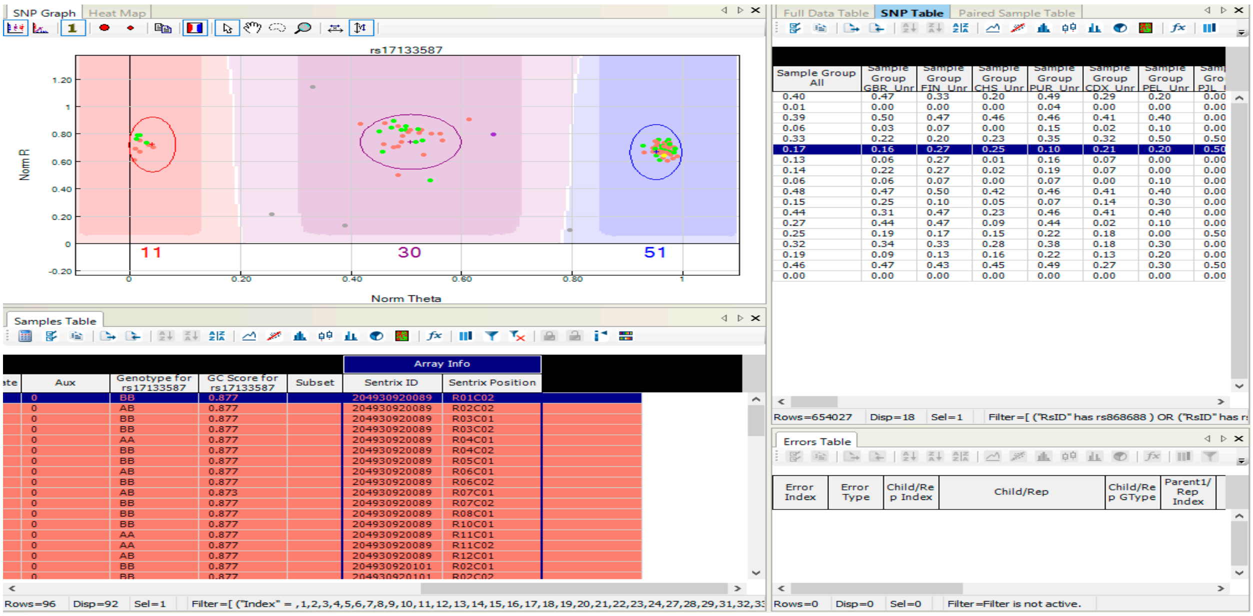

Figure S3: An example of genotyping and annotation data processing.

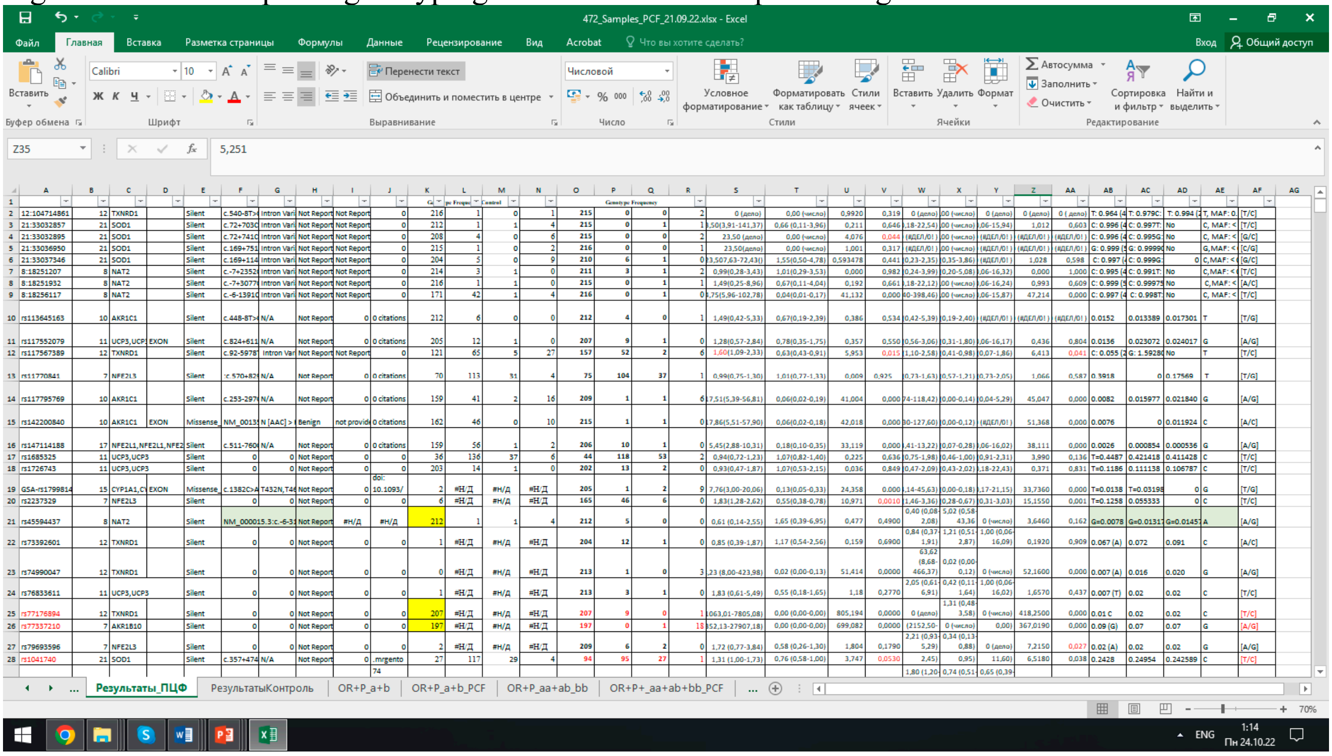

Table S3: Characteristics of functions of genes selected for study of pesticides effects on health.

Gene/Genes	Function and description
Detoxication genes	

GST genes	Glutathione S-transferases (GSTs) are important metabolic enzymes of phase II biotransformation. GSTs, including GSTM1, GSTT1, and GSTP1, play an important role in the detoxification of a wide range of xenobiotics, including carcinogens such as benzene, organochlorines, organophosphates, pesticides, tobacco smoke, chemotherapeutic agents, and reactive oxygen species. Polymorphisms of the GSTT1 and GSTP1 genes are associated with several malignancies, including leukemia and lymphoma. GSTs act by catalyzing the reaction of glutathione with an acceptor molecule to form S-substituted glutathione (S indicates sulfur). Individuals with a deletion of GSTM1 and GSTT1 may have an impaired ability to metabolically excrete organochlorine pesticides, which may affect hormonal homeostasis. This leads to an increased risk of hypospadias.
CYP1A1	CYP1A1 (aryl hydrocarbon hydroxylase) is involved in Phase I of xenobiotic and drug metabolism. The *CYP1A1* gene plays a key role in the activation of many pro-carcinogens, including polycyclic aromatic hydrocarbons and aromatic amines produced in tobacco-related products. *CYP1A1* gene is associated with the development of cancer and a number of other diseases, such as arthritis, allergies, allergic dermatitis, miscarriages, etc. The pesticide dicofol enhances the metabolism of 7-ethoxyresorufin, pentoxyresorufin, aniline, and erythromycin, which are selective substrates for CYP1A1, CYP2B, CYP2E1, and CYP3A. There is evidence of the involvement of the AhR/CYP1A1 signaling pathway in the mechanism of action of the pesticides DDT and DDE in the human placenta.
CYP2B6	Highly inducible and polymorphic enzyme that is expressed in the brain, mainly in neurons and astrocytes. It metabolizes not only clinically important drugs (e.g., bupropion, cyclophosphamide, efavirenz, propofol, and selegiline) but also many chemicals. Transcription of human CYP2B6 is directly regulated by the human pregnane X receptor (PXR). Transactivation of CYP2B6 by PXR is mediated by a region of the PBREM gene. This 51 bp amplifier module regulates constitutive androstane receptor (CAR)-mediated CYP2B6 induction. CYP2B6 has also been confirmed to be involved in the bioactivation of the pesticide chlorpyrifos (CPF). CYP2B6 variants have the potential to alter the balance of CPF metabolism and thus are likely biomarkers of susceptibility to this pesticide.
CYP2C19	Polymorphic gene with over 30 different alleles. CYP2C19 is responsible for the metabolism of 10% of drugs commonly prescribed in the clinical setting; for example, omeprazole, lansoprazole, and the anticonvulsant mephenytoin. Among all ethnic groups, Asians have a higher frequency of individuals who poorly metabolize CYP2C19. Some drugs, such as clopidogrel, are known to be less effective in some individuals belonging to Asian populations because they have a higher frequency of non-functional CYP2C19 alleles. In addition, human exposure to pesticides can provoke inhibition of this enzyme. For example, the chiral pesticide ethofumesate strongly inhibits CYP2C19 in vitro. Studies by Wendela A. Kappers et al. show that CYP2C19 is the main enzyme involved in the activation of diazinon in the human liver, while other enzymes, including CYP1A2, may play a minor role. Another pesticide, chlorpyrifos, can be

	activated to a potent β-esterase inhibitor by CYP2B6 or detoxified to dearylated metabolites by CYP2C19.
Antioxidant response genes	
GCLC and *GCLM*	Glutathione tripeptide (GSH; γ-glutamylcysteinylglycine) is one of the most distributed cellular thiols. GSH is a major player in cellular defense against ROS because it nonenzymatically scavenges both singlet oxygen and hydroxyl radicals and is used by glutathione peroxidase and glutathione transferases to limit the levels of certain reactive aldehydes and peroxides in the cell. It contains oxidative stress-sensitive elements in the promoter/enhancer region, and the GCLC gene polymorphisms associated with reduced GCLC expression are suggested to be important determinants of susceptibility to oxidative stress and DNA damage.
GPX4	Glutathione peroxidase exists in at least five isoforms in humans, GPX1-5. Of these five types, GPX4 protects cells from membrane peroxidation and cell death by catalyzing the reduction of hydrogen peroxide and lipid peroxides at the expense of reduced glutathione, thereby protecting cells from oxidative stress. GPx4 is able to regenerate complex lipid peroxides such as phospholipid hydroperoxides even when integrated into highly structured lipid-protein complexes such as lipoproteins and membranes.
SOD1	SOD plays a vital role in homeostatic redox balance by scavenging oxygen radicals and thus protecting the cell from oxidative stress. Oxidative stress caused by pesticide exposure can lead to inhibition of SOD enzyme activity, leading to accumulation of superoxide radicals, which has recently been observed among agricultural workers exposed to organophosphate, pyrethroid pesticides, and redox-cyclic herbicides. Superoxide dismutase 1 (SOD1) is one of the most important enzymes involved in the control of cellular redox homeostasis due to its ability to catalyze the dismutation of superoxide anions into hydrogen peroxide and molecular oxygen. It is associated with a number of diseases, including cardiovascular disease and Parkinson's disease.
NFE2L3	The transcription factor NFE2L3 is involved in various cellular processes, including carcinogenesis, stress response, differentiation, and inflammation.
DNA repair genes	
XRCC1 and XRCC3	The protein encoded by XRCC1 is involved in the efficient repair of DNA single-strand breaks formed by exposure to ionizing radiation and alkylating agents. This protein interacts with DNA ligase III, polymerase beta, and poly (ADP-ribose) polymerase to participate in the base excision repair pathway. XRCC3 encodes a member of the RecA/Rad51-related protein family that participates in homologous recombination to maintain chromosome stability and repair DNA damage. The polymorphism of these genes affects the activity of the proteins involved in the repair of DNA single-strand breaks, which increases the risk of developing a number of oncological diseases.

XPD	The XPD protein responsible for helicase activity is involved in DNA excision repair and transcription initiation. Mutations in the XPD gene are associated with a decrease in the efficiency of DNA damage repair.
